# Barriers and Blueprints: Next-Generation Engineering Strategies for CAR-T Cell Therapy in Gastrointestinal Tumors

**DOI:** 10.3390/ph19091449

**Published:** 2026-09-12

**Authors:** Mariam Ismail, Noran Al-Gizey, Zaid Alabed, Nour Mustafa, Ebtesam Al-Najjar, Yazan Hamdaneh, Nehal Eid, Abdullah Esmail

**Affiliations:** 1 Department of Medicine, Houston Methodist Hospital, Houston, TX 77030, USA; 2 Faculty of Medicine, Delta University for Science and Technology, Gamasa 7730103, Egypt; 3 College of Medicine, University of Baghdad, Baghdad 12114, Iraq; 4 Faculty of Medicine, The University of Jordan, Amman 11942, Jordan; 5 Department of Medicine, Methodist Healthcare System of San Antonio, San Antonio, TX 78229, USA; 6 Faculty of Medicine, Mansoura University, Mansoura 35516, Egypt; 7 Department of Medicine, Weill Cornell Medical College, New York, NY 10065, USA

**Keywords:** CAR-T cell therapy, CAR-NK cells, gastrointestinal cancers, solid tumors, tumor microenvironment, genetic engineering, precision oncology, adoptive cell therapy, immunotherapy, synthetic biology

## Abstract

Gastrointestinal (GI) malignancies account for approximately one-quarter of new cancer diagnoses and more than one-third of cancer-related deaths worldwide, yet as of August 2026, only one CAR-T therapy has received regulatory approval for a solid tumor indication anywhere in the world. The recent CT041-ST-01 phase II trial of satricabtagene autoleucel, the first randomized CAR-T trial conducted in a solid tumor, demonstrated a significant improvement in progression-free survival for patients with advanced gastric cancer (median 3.25 vs. 1.77 months; hazard ratio (HR) 0.37, *p* < 0.001). While this landmark study established the clinical feasibility of CAR-T therapy in solid tumors, it also underscored the biological barriers that continue to limit durable responses. GI tumors are characterized by heterogeneous antigen expression, dense desmoplastic stroma, inefficient immune-cell trafficking, profoundly immunosuppressive tumor microenvironments, and progressive T-cell dysfunction, all of which are further compounded by the logistical and economic challenges of autologous cell manufacturing. In this narrative review, we organize these obstacles within a unified four-barrier engineering framework and critically examine the strategies being developed to overcome each of them. We discuss advances in multi-antigen and logic-gated CAR architectures, stromal remodeling through fibroblast activation protein (FAP)-targeted approaches and extracellular matrix-degrading enzymes, chemokine receptor engineering, regional delivery, hypoxia-responsive CARs, cytokine-armored and persistence-enhanced constructs, dominant-negative and switch receptors, metabolic reprogramming, and intrinsic checkpoint disruption. We also review emerging manufacturing platforms, including allogeneic CAR-T and CAR-natural killer (CAR-NK) cells, induced pluripotent stem cell-derived products, CAR-macrophages, and in vivo CAR generation, together with engineering strategies designed to improve safety and scalability. Rather than relying on a single technological advance, the future of CAR-based therapy for GI malignancies will likely depend on integrating multiple engineering approaches to address the diverse biological barriers within the tumor microenvironment. By synthesizing current preclinical and early clinical evidence, this review provides a translational framework for the next generation of CAR-based cellular therapies in gastrointestinal oncology.

## 1. Introduction

### 1.1. The Unmet Need

Gastrointestinal (GI) cancers, including malignancies of the esophagus, stomach, colorectum, liver, pancreas, and biliary tract, remain a major global health challenge. Collectively, they account for approximately one-quarter of all newly diagnosed cancers and more than one-third of cancer-related deaths worldwide [1]. Despite advances in prevention, diagnosis, and systemic therapy, the global burden continues to rise. In 2023, colorectal cancer accounted for approximately 2.29 million new cases and 1.11 million deaths, stomach cancer for 1.26 million cases and 935,000 deaths, and liver cancer for 570,000 new diagnoses while maintaining one of the highest mortality-to-incidence ratios among solid tumors [1]. Pancreatic cancer likewise remains one of the deadliest human malignancies [1]. GLOBOCAN 2022 projects that annual GI cancer cases will reach 9.06 million and deaths 6.42 million by 2050, with the greatest increases expected in countries with low and middle Human Development Index [2].

Poor outcomes largely reflect late diagnosis and the limited efficacy of current systemic therapies. Mortality-to-incidence ratios approach 0.95 for esophageal, liver, and pancreatic cancers, underscoring their aggressive biology and the lack of effective screening strategies [3]. Although screening has reduced mortality from colorectal cancer, it remains a leading cause of cancer-related death worldwide, and its rising incidence among younger adults has emerged as an additional concern [2].

Immune checkpoint inhibitors (ICIs) have improved outcomes for selected molecular subgroups, including microsatellite instability-high (MSI-H) or mismatch repair-deficient (dMMR) tumors, and are now incorporated into first-line treatment for selected gastric, esophageal, and hepatocellular cancers [4,5]. However, these benefits are limited to a minority of patients. More than 90% of GI cancers are microsatellite stable (MSS), in which response rates to ICI monotherapy remain below 10% [6]. Even combinations of ICIs with tyrosine kinase inhibitors have produced only modest activity in MSS colorectal cancer, with pooled objective response rates of approximately 12% and a median progression-free survival of 3.5 months [7]. These limited responses largely reflect the immunologically “cold” tumor microenvironment of most GI malignancies, characterized by low tumor mutational burden, poor T-cell infiltration, and abundant immunosuppressive stromal and myeloid cells [8]. Consequently, increasing attention has shifted toward adoptive cellular therapies capable of generating tumor-specific immune responses rather than simply restoring endogenous immunity.

Recent clinical progress has reinforced this shift. In the randomized phase II CT041-ST-01 trial, patients with claudin 18.2 (CLDN18.2)-positive advanced gastric or gastroesophageal junction adenocarcinoma treated with satricabtagene autoleucel (satri-cel) achieved a median progression-free survival of 3.25 months compared with 1.77 months with physician’s choice therapy (Hazard Ratio (HR) 0.37, *p* < 0.001) [9]. As the first randomized trial to demonstrate a clinical benefit for CAR-T cell therapy in a solid tumor, CT041-ST-01 represents an important proof of concept. Nevertheless, the modest absolute improvement in disease control highlights the need for engineering strategies capable of overcoming the multiple biological barriers that continue to limit durable responses in GI solid tumors.

### 1.2. CAR Biology Primer

Chimeric antigen receptor (CAR) T-cell therapy redirects T lymphocytes toward predefined tumor antigens by introducing a synthetic receptor that recognizes surface targets independently of major histocompatibility complex (MHC) presentation. The CAR consists of an extracellular antigen-binding domain, most commonly a single-chain variable fragment (scFv), linked through a hinge and transmembrane region to intracellular signaling domains comprising CD3ζ and one or more costimulatory molecules [10,11,12]. In current clinical practice, autologous T cells are collected by leukapheresis, genetically modified ex vivo using viral vectors, expanded, and reinfused following lymphodepleting chemotherapy [10,11].

Progressive refinements in CAR design have focused on improving T-cell activation, persistence, and durability. First-generation CARs incorporated CD3ζ alone and mediated antigen-specific cytotoxicity but showed limited in vivo expansion [13,14]. The addition of a single costimulatory domain in second-generation constructs markedly improved clinical activity. CD28-based CARs promote rapid effector expansion, whereas 4-1BB-containing constructs favor memory formation and prolonged persistence [12,15]. As of August 2026, all currently FDA-approved CAR-T products use second-generation designs [10,15]. Third-generation CARs combine two costimulatory domains, although consistent clinical advantages over second-generation constructs have not been established [10,16]. More recent platforms have expanded CAR function beyond cytotoxicity: fourth-generation CARs, termed TRUCKs (T cells Redirected for Universal Cytokine-mediated Killing), incorporate inducible cytokine payloads such as IL-12, IL-15, or IL-21 to remodel the tumor microenvironment, whereas fifth-generation constructs integrate a truncated IL-2 receptor β domain to activate JAK-STAT3/5 signaling and enhance proliferation and persistence [17,18].

The success of CAR-T therapy in hematologic malignancies established adoptive cell therapy as a new therapeutic paradigm. Durable remissions have been achieved in pediatric and young adult B-cell acute lymphoblastic leukemia with tisagenlecleucel [19], relapsed or refractory large B-cell lymphoma with axicabtagene ciloleucel [20], and multiple myeloma with idecabtagene vicleucel [21]. As of August 2026, six CAR-T products are approved by the U.S. Food and Drug Administration (FDA) across B-cell leukemias, lymphomas, chronic lymphocytic leukemia, and multiple myeloma [10,15]. In contrast, no CAR-T therapy has yet received FDA approval for a solid tumor; however, in June 2026, China’s National Medical Products Administration (NMPA) approved satricabtagene autoleucel for advanced gastric/gastroesophageal junction cancer, marking the world’s first regulatory approval of a CAR-T therapy for a solid tumor and underscoring the rapidly evolving pace of the field [10,22,23].

### 1.3. The Four-Barrier Framework and Scope

The limited efficacy of conventional CAR-T cells in GI solid tumors reflects the convergence of several biological barriers rather than a single dominant mechanism. Throughout this review, these challenges are discussed within a four-barrier framework encompassing antigen targeting, tumor trafficking, the immunosuppressive microenvironment, and T-cell dysfunction [8,10,24,25].

#### 1.3.1. Barrier 1: Antigen Selection and Targeting Fidelity

In contrast to hematologic malignancies, where targets such as CD19 and B-cell maturation antigen (BCMA) are broadly and selectively expressed, GI tumors lack an ideal surface antigen. Leading candidates, including CLDN18.2, mesothelin, glypican-3 (GPC3), carcinoembryonic antigen (CEA), HER2, epithelial cell adhesion molecule (EpCAM), and MUC1, display variable expression both between and within tumors and are often shared with normal tissues. As a result, CAR-T therapies face the dual challenge of antigen escape and on-target, off-tumor toxicity [24,26].

#### 1.3.2. Barrier 2: Trafficking and Infiltration

Following intravenous infusion, CAR-T cells must reach the tumor through a series of sequential steps that include vascular adhesion, transendothelial migration, chemokine-guided homing, and penetration of the extracellular matrix. Each step is impaired in GI malignancies by abnormal tumor vasculature, dense stromal architecture, and mismatched chemokine signaling, with the greatest obstacles encountered in highly desmoplastic tumors such as pancreatic ductal adenocarcinoma [25,27,28].

#### 1.3.3. Barrier 3: The Immunosuppressive Tumor Microenvironment

Even when CAR-T cells successfully infiltrate the tumor, they encounter an environment dominated by suppressive immune populations, inhibitory checkpoint ligands, soluble immunomodulatory factors, hypoxia, and nutrient deprivation, all of which impair effector function [8,24]. Cancer-associated fibroblasts further reinforce this barrier by contributing to both stromal remodeling and active immune suppression [10,28].

#### 1.3.4. Barrier 4: T-Cell Exhaustion and Limited Persistence

Sustained antigen exposure within the tumor microenvironment progressively drives CAR-T cells toward an exhausted state characterized by inhibitory receptor expression, epigenetic reprogramming, metabolic dysfunction, and loss of cytotoxic capacity [8,29]. Antigen-independent tonic signaling can accelerate this process, while the metabolic constraints imposed by the tumor microenvironment further compromise long-term persistence [10,12].

Although presented separately, these barriers are tightly interconnected. Limited trafficking restricts CAR-T cells to stromal-rich regions, where immunosuppressive signals accelerate exhaustion. At the same time, antigen heterogeneity allows resistant tumor clones to persist even when functional CAR-T cells successfully infiltrate the tumor. Overcoming any single obstacle is therefore unlikely to be sufficient, highlighting the need for integrated engineering strategies that address multiple barriers simultaneously.

**Figure 1:**
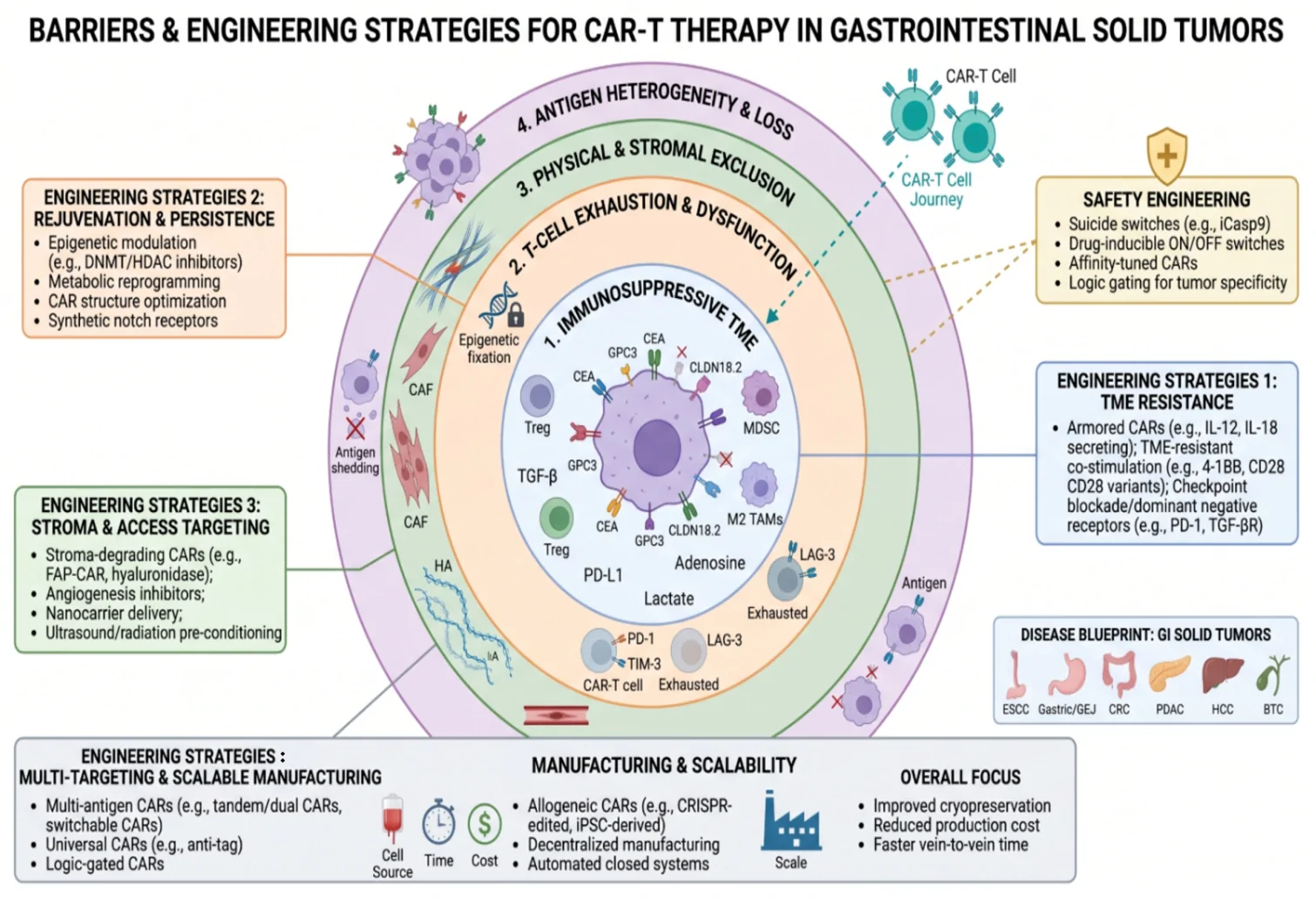
Four-Barrier Framework for CAR-Based Cell Therapy in GI Solid Tumors.

This review focuses on CAR-based cellular therapies, including CAR-T, CAR-NK, and CAR-macrophage platforms, for esophageal, gastric and gastroesophageal junction, colorectal, pancreatic, hepatocellular, and biliary tract cancers. Because clinical development remains largely confined to early-phase studies, with most trials originating from China and the United States [24,25], we emphasize the biological rationale underlying each barrier, critically evaluate the engineering strategies designed to overcome it, and summarize the available preclinical and clinical evidence. We conclude by discussing emerging combinatorial approaches, manufacturing challenges, and future directions for clinical translation (Figure 1).

### 1.4. Literature Search Strategy

To identify relevant studies for this review, a systematic search of the literature was conducted across major electronic databases, including PubMed, Scopus, and Web of Science, alongside ClinicalTrials.gov to capture completed and ongoing clinical trials. The search period spanned from database inception through August 2026, ensuring the inclusion of highly contemporary preclinical and clinical breakthroughs in the rapidly evolving field of adoptive cellular therapies. As this is a narrative rather than a systematic review, the search strategy was not designed to comply with PRISMA (Preferred Reporting Items for Systematic Reviews and Meta-Analyses) reporting standards; rather, it was structured to ensure comprehensive, rigorous, and current coverage of a rapidly evolving field.

The search strategy utilized combinations of Medical Subject Headings (MeSH) terms and free-text keywords, including: “chimeric antigen receptor”, “CAR-T”, “CAR-NK”, “adoptive cell therapy”, “synthetic biology”, “genetic engineering”, “gastrointestinal oncology”, “gastric cancer”, “colorectal cancer”, “pancreatic cancer”, “hepatocellular carcinoma”, “tumor microenvironment”, “logic gates”, “Tmod”, “allogeneic”, “in vivo CAR”, and “safety switches”.

Studies were included if they investigated chimeric antigen receptor (CAR)-based platforms (including CAR-T, CAR-NK, CAR-macrophages, and engineered multi-lineage constructs) targeting gastrointestinal solid tumors. Only studies published in the English language were eligible. Studies focusing solely on hematologic malignancies, non-CAR immunotherapies, or those lacking clear translational relevance to gastrointestinal malignancies were excluded. The search strategy prioritized full-text, peer-reviewed original research and review articles. Conference and meeting abstracts were strictly minimized, utilized only when representing highly critical, newly disclosed clinical trial data, and systematically updated to full-text publications where available.

Preclinical evidence (comprising in vitro assays, 3D tumor spheroids, patient-derived organoids, and in vivo xenograft or syngeneic mouse models) was systematically evaluated to elucidate the mechanistic underpinnings of next-generation engineering strategies. Clinical evidence (from early-phase trials) was critically appraised and prioritized to evaluate translationally crucial outcomes, including objective response rate (ORR), progression-free survival (PFS), dose-limiting toxicities, and the duration of clinical responses, thereby highlighting the biological bottlenecks that currently distinguish laboratory models from real-world patient outcomes.

## 2. The Barrier Landscape

Translational progress of CAR-based cell therapies in gastrointestinal (GI) solid tumors remains limited by the convergence of four distinct biological barriers: target antigen heterogeneity, physical stromal exclusion, microenvironmental immunosuppression, and T-cell exhaustion. Crucially, while Section 2.1, Section 2.2, Section 2.3 and Section 2.4 systematically evaluate these intrinsic microenvironmental hurdles, Section 2.5 addresses the extrinsic logistical, temporal, and financial constraints of cell manufacturing. We conceptually define these manufacturing variables as an overarching translational bottleneck that structurally frames the therapeutic viability of the biological strategies discussed.

### 2.1. Antigen Heterogeneity and Escape

The remarkable clinical success of CAR-T therapy in B-cell malignancies is largely attributable to the biology of its targets. CD19 and BCMA are expressed uniformly across the malignant lineage while remaining dispensable in normal tissues apart from predictable B-cell depletion, allowing highly effective tumor eradication with clinically manageable toxicity [10]. Nevertheless, even under these favorable conditions, antigen escape remains a major mechanism of treatment failure. CD19 loss accounts for approximately 28% of relapses following axicabtagene ciloleucel in large B-cell lymphoma and up to 91% of relapses after tisagenlecleucel in B-cell acute lymphoblastic leukemia, highlighting the inherent vulnerability of single-antigen targeting [10].

This challenge is substantially greater in GI malignancies, where no equivalent tumor-specific antigen exists. Candidate targets are heterogeneous both across patients and within individual tumors, and most retain measurable expression in normal tissues [24,26]. Expression also varies considerably among targets. GPC3 is detected in approximately 70–80% of hepatocellular carcinomas [30,31], mesothelin in 80–100% of pancreatic ductal adenocarcinomas [32,33], and GUCY2C in more than 95% of well-differentiated colorectal cancers [34]. In contrast, HER2 amplification is observed in only 17–20% of gastric cancers and 3–6% of colorectal cancers [35,36], while CLDN18.2 reaches therapeutically relevant expression in only 40–60% of gastric adenocarcinomas and is constitutively expressed in normal gastric mucosa [37]. This overlap with healthy tissues narrows the therapeutic window. Accordingly, CLDN18.2-directed CAR-T cells have produced dose-dependent gastric mucosal injury in preclinical studies, and gastrointestinal toxicities consistent with on-target, off-tumor activity were reported in the phase I satricabtagene autoleucel (satri-cel) trial [38,39]. Similar limitations apply to mesothelin, which is expressed on normal mesothelial surfaces, and CEA, which is present in normal colonic epithelium [24,26].

Tumors further evade CAR-T cell therapy through dynamic changes in antigen expression. Reported mechanisms include transcriptional downregulation, complete antigen loss, alternative splicing that removes the CAR-binding epitope, masking by soluble or extracellular matrix-associated proteins, and trogocytosis, in which antigen transfer from tumor cells to CAR-T cells simultaneously reduces tumor antigen density and promotes CAR-T fratricide [40,41]. Several of these mechanisms are particularly relevant in GI cancers. Mesothelin undergoes proteolytic shedding, generating soluble mesothelin-related peptide that can bind and sequester CARs before they reach tumor cells [42]. GPC3 shedding has also been reported in hepatocellular carcinoma [43]. More recently, IFNγ released by activated CAR-T cells was shown to suppress target antigen expression through JAK-STAT signaling, creating an adaptive feedback mechanism that promotes immune escape [44].

Not all antigens are equally susceptible to immune escape. The consequences of antigen loss depend on the biological function of the target itself. GPC3 contributes directly to hepatocellular carcinoma progression through activation of canonical Wnt signaling, making its loss biologically costly for tumor cells [45]. By contrast, CEA primarily functions as an adhesion molecule, while GUCY2C acts as a tumor suppressor whose ligand guanylin is already universally silenced in colorectal cancer. Downregulation of these targets therefore imposes little, if any, fitness penalty and may even confer a selective advantage under immune pressure [46,47]. Consequently, the biological importance of a target may be as critical as its expression profile when selecting antigens for CAR-based therapy.

These factors collectively explain why antigen-negative relapse remains a recurring pattern even after initial clinical responses. Such escape has already been documented in patients treated with CLDN18.2-directed CT041 and GCC-targeted CAR-T therapies, underscoring the need for strategies that extend beyond single-antigen targeting [39,48].

Here is a case study regarding the mechanistic basis for Cldn18.2 CAR-T clinical efficacy. The regulatory approval of satricabtagene autoleucel (satri-cel) in June 2026 by China’s National Medical Products Administration (NMPA) represents a milestone in cancer immunotherapy, establishing the first approved CAR-T cell therapy for a solid tumor indication (24). Specifically indicated for CLDN18.2-positive, HER2-negative advanced gastric or gastroesophageal junction adenocarcinoma after failure of at least two prior lines of therapy, satri-cel is clinically anchored in the randomized phase II CT041-ST-01 trial, which demonstrated a significant improvement in progression-free survival (Section 1.1), corroborated by final phase I trial results showing durable responses and a manageable toxicity profile [39]. Understanding why this platform succeeded where earlier solid-tumor CAR-T strategies did not requires considering several converging biological, clinical, and pharmacological factors.

A central contributor to satri-cel’s success is the lineage-restricted accessibility of the CLDN18.2 antigen. In healthy tissue, CLDN18.2 expression is confined to differentiated gastric mucosal epithelial cells, where the protein is physically sequestered within tight junctions and largely inaccessible to circulating CAR-T cells; malignant transformation disrupts this polarity and exposes the epitope, conferring a degree of tumor selectivity despite shared lineage expression. As discussed above, CLDN18.2 reaches therapeutically relevant expression in a substantial proportion of gastric adenocarcinomas, sufficient in these patients to support robust CAR engagement; however, this expression remains heterogeneous both across and within tumors and does not eliminate the risk of antigen-negative relapse. Consistent with this dual profile, CLDN18.2-directed CAR-T cells have produced dose-dependent gastric mucosal injury in preclinical models [38], and transient gastrointestinal toxicities consistent with on-target, off-tumor activity were reported in the phase I trial, though these adverse events proved clinically manageable [39].

Beyond antigen selection, satri-cel’s clinical activity has been attributed to modification of the standard preconditioning regimen. Rather than fludarabine/cyclophosphamide lymphodepletion alone, later protocol iterations reportedly incorporated low-dose nab-paclitaxel before CAR-T infusion, intended to disrupt desmoplastic stroma and enhance CAR-T trafficking and intratumoral infiltration.

Clinical experience with the CT041 program further indicates that treatment sequencing and baseline T-cell fitness materially affect outcomes. Administering satri-cel earlier in the treatment course, rather than as late-stage salvage therapy in heavily pretreated, immune-depleted patients, is associated with greater lymphocyte proliferative capacity and reduced terminal differentiation, translating into superior CAR-T expansion and more durable responses [49].

Collectively, these observations indicate that satri-cel’s clinical validation reflects a favorable convergence of lineage-restricted antigen biology, pharmacological modification of the stromal barrier, and optimized treatment timing, rather than resolution of any single barrier in isolation, reinforcing this review’s central argument that durable solid-tumor CAR-T efficacy will likely require coordinated engineering across all four barriers simultaneously.

### 2.2. Physical and Stromal Barriers

Unlike hematologic malignancies, where CAR-T cells encounter their targets within the circulation, bone marrow, and lymphoid tissues, GI solid tumors present substantial anatomical barriers to immune-cell trafficking. Following intravenous infusion, CAR-T cells must survive systemic distribution, adhere to tumor endothelium, extravasate into the tumor bed, migrate through a dense stromal compartment, and ultimately reach malignant cells. Inefficiency at any stage markedly reduces the number of effector cells reaching the tumor [8,28].

Tumor vasculature in GI cancers is highly abnormal. VEGF-driven angiogenesis produces tortuous, poorly organized vessels with limited pericyte coverage and elevated interstitial fluid pressure, all of which impair transendothelial migration [8]. Endothelial cells further limit immune-cell recruitment by downregulating adhesion molecules such as ICAM-1 and VCAM-1 [28]. In parallel, mismatches between chemokines produced within the tumor and the receptors expressed by infused CAR-T cells substantially reduce directional homing [28,50].

After crossing the vascular barrier, CAR-T cells encounter a dense stromal network composed of cancer-associated fibroblasts (CAFs), extracellular matrix proteins, including collagen, hyaluronic acid, and fibronectin, and mesenchymal stromal cells that collectively restrict immune-cell migration [8,28]. This barrier is particularly pronounced in pancreatic ductal adenocarcinoma (PDAC), where desmoplastic stroma can account for 80–90% of the tumor mass [27,51]. Beyond their structural role, CAFs actively suppress antitumor immunity by secreting TGF-β, IL-6, and CXCL12, recruiting regulatory immune cells, and upregulating PD-L1 [8,52]. These observations have driven the development of stromal-directed approaches, including FAP-targeted CAR-T cells and heparanase-expressing constructs designed to improve intratumoral trafficking [53,54].

### 2.3. Immunosuppressive Tumor Microenvironment

Even when CAR-T cells successfully infiltrate GI tumors, their activity is rapidly curtailed by a profoundly immunosuppressive microenvironment shaped by cellular, soluble, and metabolic factors [8,24].

Regulatory T cells (Tregs), myeloid-derived suppressor cells (MDSCs), and tumor-associated macrophages (TAMs) dominate the immune landscape of many GI malignancies and frequently outnumber cytotoxic lymphocytes. Tregs suppress CAR-T function through CTLA-4-mediated competition for costimulatory ligands, secretion of IL-10 and TGF-β, and generation of adenosine via the CD39/CD73 pathway [8]. MDSCs inhibit T-cell proliferation through arginase, inducible nitric oxide synthase, and reactive oxygen species, whereas M2-polarized TAMs recruit additional Tregs through CCL22, reinforce stromal remodeling, and express PD-L1, further suppressing CAR-T activity [8,28].

Soluble immunosuppressive mediators amplify these effects. TGF-β, which is highly expressed in pancreatic and gastric cancers, directly inhibits CAR-T cytotoxicity while promoting differentiation toward an induced regulatory phenotype [51]. Tumor and stromal cells also express checkpoint ligands, including PD-L1, galectin-9, and CEACAM1, that engage inhibitory receptors on CAR-T cells and dampen effector function [8,24].

Metabolic stress further limits CAR-T cell persistence. Hypoxia, glucose deprivation, depletion of amino acids such as tryptophan and arginine, and accumulation of lactate, kynurenine, and adenosine collectively impair mitochondrial fitness, suppress mTOR signaling, and restrict T-cell proliferation [8,25].

### 2.4. T-Cell Exhaustion and Limited Persistence

Persistent antigen exposure within the immunosuppressive tumor microenvironment drives progressive expression of inhibitory receptors, including PD-1, LAG-3, TIM-3, and TIGIT, together with TOX- and NR4A-dependent epigenetic remodeling, culminating in durable loss of proliferative capacity and cytotoxic function [55,56,57]. Unlike the transient dysfunction observed during acute infection, this exhausted state becomes epigenetically fixed and is only partially reversible with checkpoint inhibition alone [8,55]. In GI solid tumors, exhaustion is compounded by the metabolic constraints described above: hypoxia, nutrient deprivation, and acidosis impair the mitochondrial fitness required for sustained effector function, while antigen-independent tonic signaling from the CAR construct itself can accelerate the transition toward terminal exhaustion even before tumor encounter [10,12]. Together, these processes explain the limited persistence observed in most early-phase CAR-T trials in GI malignancies and underscore the need for engineering strategies that maintain durable effector function.

### 2.5. Overarching Translational Bottleneck: Manufacturing and Scalability Constraints

The challenges facing CAR-based therapies extend beyond tumor biology. The current autologous manufacturing model introduces substantial logistical, temporal, and economic barriers that become even more pronounced in patients with advanced GI malignancies. Manufacturing FDA-approved CAR-T products requires leukapheresis, ex vivo T-cell activation, viral vector transduction, expansion, quality-control testing, and cryopreservation before infusion, resulting in vein-to-vein intervals of approximately 13–54 days [10]. During this period, 0–31% of patients fail to receive their intended therapy, most often because of rapid disease progression rather than manufacturing failure [10].

These delays are especially problematic in pancreatic, gastric, and hepatocellular cancers, where clinical deterioration can occur within weeks. Consequently, T-cell collection is increasingly being performed before or early during first-line treatment, when lymphocytes retain greater proliferative capacity and less terminal differentiation. In contrast, repeated exposure to cytotoxic chemotherapy reduces T-cell fitness, limiting ex vivo expansion and long-term persistence after infusion [10,15]. Clinical experience with the CT041 program supports this concept, as patients treated with satricabtagene autoleucel (satri-cel) after first-line chemotherapy achieved superior CAR-T expansion and more durable responses than heavily pretreated patients [49].

Manufacturing costs remain another major obstacle. Current FDA-approved CAR-T therapies are priced at several hundred thousand dollars per infusion, while individualized manufacturing limits production capacity and prevents the economies of scale achievable with conventional pharmaceuticals [10]. This limitation is particularly relevant in GI oncology, where the number of potentially eligible patients far exceeds that of current hematologic indications.

## 3. Precision Antigen Targeting: Multi-Antigen and Logic-Gated Architectures

### 3.1. Rationale for Multi-Antigen Targeting in GI Tumors

Single-antigen CAR strategies face a fundamental limitation in gastrointestinal (GI) solid tumors: no available target is expressed uniformly enough, or selectively enough, to ensure complete tumor eradication without risking normal-tissue toxicity. Unlike CD19 or BCMA in hematologic malignancies, GI tumor antigens are often variably expressed across patients, unevenly distributed within individual tumors, and shared to some extent with normal epithelial tissues. CLDN18.2 is expressed at therapeutically relevant levels in approximately 40–60% of gastric adenocarcinomas, GPC3 in 70–80% of hepatocellular carcinomas, and HER2 in only 17–20% of gastric cancers and 3–6% of colorectal cancers [24,26]. Even GUCY2C, one of the most broadly expressed colorectal cancer targets, is less consistently maintained in metastatic disease than in primary tumors [34,46]. This heterogeneity creates a reservoir of antigen-low or antigen-negative cells that can survive selective CAR pressure and drive relapses.

Experience from hematologic malignancies underscores this risk. Antigen-loss relapse occurs even when the initial target is highly homogeneous, as illustrated by CD19-negative relapse after CD19-directed CAR-T therapy in B-cell acute lymphoblastic leukemia and large B-cell lymphoma [10]. In GI cancers, where antigen expression is heterogeneous at baseline, the evolutionary pressure imposed by single-target therapy is likely to accelerate the emergence of resistant clones.

Target selection should therefore consider not only antigen prevalence but also the biological cost of antigen loss. Some targets, such as GPC3, actively support tumor growth through oncogenic signaling pathways, making complete loss potentially disadvantageous to the tumor cell [45]. Others, such as CEA or GUCY2C, may be more dispensable in established malignancy, allowing tumor cells to downregulate expression with limited fitness penalty [46,47]. Consequently, multi-antigen strategies should combine targets with complementary expression patterns and distinct escape liabilities, thereby increasing the evolutionary cost of immune evasion.

### 3.2. Tandem and Bivalent CARs

The most direct approach to multi-antigen targeting is to incorporate two antigen-binding domains into a single CAR construct. These tandem or bivalent CARs usually function as OR-gates, meaning that recognition of either antigen can trigger activation. This design broadens tumor coverage while preserving the practical advantage of a single engineered cell product [11,58].

The most clinically advanced example in GI oncology is KD-496, a bispecific CAR-T product targeting CLDN18.2 and Natural Killer Group 2D (NKG2D) ligands. Early phase I data presented in treatment-refractory GI cancers showed encouraging activity, with responses observed in both gastric and pancreatic cancer cohorts [28]. Importantly, severe gastrointestinal toxicity was not reported, a notable finding given the concern for on-target, off-tumor injury with CLDN18.2-directed therapies. Although these data remain preliminary and involve a small cohort, KD-496 provides proof of concept that dual-antigen recognition may improve tumor coverage while maintaining a manageable safety profile.

Several preclinical tandem designs further support this principle. A PD-L1/CLDN18.2 bicistronic CAR showed stronger tumor inhibition than a single CLDN18.2 CAR in gastric cancer models, with evidence of improved expansion and reduced exhaustion-associated signatures [59]. MSLN/MUC16 tandem CARs demonstrated antigen-density-dependent killing and outperformed monospecific constructs in mixed-antigen tumor models [60]. In hepatocellular carcinoma, GPC3/CD133 bispecific CARs improved tumor control compared with single-target approaches [61]. Similarly, CD70/B7-H3 tandem CARs showed enhanced cytotoxicity in esophageal, liver, and colorectal cancer models with confirmed antigen co-expression [62].

Despite these advantages, tandem CARs introduce design constraints. Larger transgenes may reduce viral vector efficiency, particularly when multiple scFvs, linkers, and signaling modules are incorporated into a single construct. Construct geometry, linker length, antigen-binding domain order, and affinity must also be optimized to minimize tonic signaling and preserve antigen-specific activation [12]. Recent manufacturing advances, including optimized workflows for large lentiviral constructs, may partially address these limitations and expand the feasibility of more complex tandem and logic-gated designs [63].

### 3.3. Co-Administration, Pooled, and Sequential Strategies

An alternative to encoding multiple specificities into one construct is to combine separate CAR-cell populations. This can be achieved through co-administration, pooled infusion, or sequential treatment. These approaches preserve construct simplicity and allow dose adjustment between cell populations, but they also increase manufacturing and regulatory complexity.

The most compelling clinical example is GCC19CART, a CoupledCAR platform that combines GCC-directed CAR-T cells with CD19-directed CAR-T cells in metastatic colorectal cancer. The CD19-directed component is intended to engage normal B cells and provide cytokine support that enhances expansion of the GCC-targeting population. In early-phase studies, GCC19CART has produced notable responses in refractory metastatic colorectal cancer, including dose-dependent activity and encouraging progression-free survival signals [64,65,66]. Although the precise contribution of the CD19-directed component remains incompletely defined, these data suggest that “helper” CAR populations may improve the performance of solid-tumor CAR-T therapy.

Sequential strategies offer a different rationale: modifying the tumor environment before delivering the tumor-directed CAR. For example, FAP-directed CAR-T cells administered before CLDN18.2 CAR-T cells depleted stromal elements, reduced suppressive myeloid recruitment, and improved CD8-positive T-cell survival in pancreatic cancer models [67]. This approach links antigen targeting directly to stromal remodeling and illustrates how multi-step cellular therapy could address more than one barrier simultaneously.

However, not all combination approaches are successful. A dual MSLN/CD19 co-administration strategy demonstrated limited persistence and only modest clinical benefit, emphasizing that simply combining two CAR products is insufficient without careful attention to expansion, persistence, trafficking, and the biological role of each cell population [68]. Overall, co-administered and sequential platforms are attractive because of their modularity, but their clinical translation will require standardized dosing strategies, synchronized manufacturing, and clear mechanistic justification.

### 3.4. Logic-Gated CAR Platforms

Logic-gated CARs extend multi-antigen targeting beyond simple antigen broadening. Rather than activating in response to either antigen, these systems use Boolean logic to improve tumor selectivity, reduce off-tumor toxicity, and restrict effector function to cells or tissues with a defined antigenic pattern. In GI cancers, where many candidate antigens are shared with normal epithelium, this added layer of control is particularly relevant.

#### 3.4.1. SynNotch Systems: IF-THEN Gates

Synthetic Notch, or synNotch, receptors use one antigen as a priming signal to induce expression of a CAR against a second antigen. Recognition of antigen A triggers receptor cleavage and release of a transcription factor, which then drives expression of a CAR targeting antigen B. This creates an IF-THEN circuit in which full cytotoxicity depends on spatial proximity of both antigens [69,70].

In hepatocellular carcinoma, GPC3-synNotch-inducible CD147 CAR-NK cells improved specificity by requiring dual-antigen recognition for full activation, reducing the risk of off-tumor cytotoxicity [11]. SynNotch systems also offer additional advantages: the CAR is not constitutively expressed, which may reduce tonic signaling; activation can be spatially restricted to the tumor site; and engineered cells may preserve a less differentiated phenotype before antigen encounter [70]. However, these benefits come at the cost of slower activation kinetics, dependence on transcription and translation, potential immunogenicity of synthetic components, and possible background signaling.

A major technical advance is the development of single-vector synNotch systems. Rommel et al. reported a single-vector synNotch architecture that integrates all required components into one lentiviral construct, reducing the need for dual transduction. In HER2-MSLN models, this design achieved selective killing of double-positive tumors and improved in vivo efficacy [63]. Beyond direct antigen gating, synNotch circuits can also be used to induce cytokine programs. For example, synNotch-induced IL-2 production enhanced CAR-T infiltration into immune-excluded tumors without causing systemic IL-2 toxicity, a concept highly relevant to microsatellite-stable colorectal cancer and pancreatic ductal adenocarcinoma [71].

#### 3.4.2. AND-Gate CARs

AND-gate CARs require simultaneous recognition of two antigens for full activation. This architecture is particularly attractive when neither antigen alone is sufficiently tumor-specific, but their co-expression pattern is enriched in malignant tissue.

The LINK CAR platform represents an important advance in this area. Instead of relying on conventional CD3ζ signaling, LINK CARs distribute proximal signaling components across two receptors, such as LAT and SLP-76. Productive T-cell activation occurs only when both receptors are engaged, and the signaling components are brought into proximity [72]. This design creates a rapid and reversible AND-gate with strong discriminatory capacity. In GI cancers, the platform could theoretically pair a broadly expressed epithelial antigen, such as CEA or EpCAM, with a more tumor-restricted antigen, such as CLDN18.2 or GPC3, to improve selectivity while preserving potency.

A more GI-specific example is OriC613, an MSLN/CLDN18.2 dual-target CAR designed to require co-expression of both antigens. By combining a high-affinity CLDN18.2-binding domain with a moderate-affinity MSLN nanobody, OriC613 achieved selective activation against double-positive tumor cells while sparing CLDN18.2-only cells in preclinical models [73]. This is clinically relevant because CLDN18.2 is expressed in normal gastric mucosa, and single-target CLDN18.2 CAR-T therapy has raised concerns about gastric toxicity [38]. Although still preclinical, OriC613 illustrates how AND-gate design may preserve antitumor activity while narrowing the therapeutic window problem that has limited several GI targets.

#### 3.4.3. NOT-Gate and Inhibitory CARs

NOT-gate systems are designed to prevent CAR activation against normal tissues. In this strategy, an activating CAR recognizes a tumor-associated antigen, while an inhibitory CAR recognizes an antigen present on normal cells. Engagement of the inhibitory receptor delivers a suppressive signal through intracellular domains such as PD-1, CTLA-4, or leukocyte immunoglobulin-like receptor 1 (LIR-1), overriding activation and protecting normal tissue [49].

Bassan et al. demonstrated this principle using a HER2 activating CAR paired with an HLA-A02-directed LIR-1-based inhibitory CAR, which protected HLA-A02-positive cells in 3D spheroid models [74]. For GI tumors, inhibitory CARs could theoretically protect normal epithelial tissues that express low levels of CEA, EpCAM, or other shared antigens. However, several challenges remain, including the timing of inhibitory signaling, the risk of incomplete protection during rapid cytotoxic activation, and the need to avoid cis-binding when inhibitory ligands are expressed by the engineered T cells themselves [75].

### 3.5. Tmod Platforms

The Tmod platform is among the most clinically advanced logic-gated systems for solid tumors. Unlike conventional NOT-gates that rely on differential antigen expression, Tmod exploits loss of heterozygosity (LOH), an irreversible genomic alteration common in cancer.

Tmod cells co-express two receptors: an activating CAR targeting a tumor-associated antigen, such as mesothelin, CEA, or EGFR, and a blocker receptor targeting HLA-A02. Normal tissues retain HLA-A02 and therefore activate the blocker, which suppresses CAR-mediated killing. Tumor cells that have lost HLA-A*02 through LOH lack the blocker ligand and are selectively eliminated [76]. This design is conceptually powerful because LOH is less easily reversed than antigen downregulation. At the same time, blocker performance depends on receptor geometry, target height, hinge length, and the balance between activating and inhibitory signals [77].

A2B694, a mesothelin-targeting Tmod product, is being evaluated in EVEREST-2. Early clinical data across solid tumors, including GI-relevant indications, showed no dose-limiting toxicities and evidence of tumor infiltration in available biopsies [78]. Although the most striking response reported to date occurred in non-small-cell lung cancer, the result supports the biological validity of the platform and is relevant to ongoing development in colorectal, pancreatic, and gastroesophageal cancers.

A2B543 builds on the A2B694 backbone by adding an inducible membrane-tethered IL-12 module under nuclear factor of activated T cells (NFAT) promoter control. This design combines logic-gated specificity with cytokine armoring, aiming to improve persistence and local immune activation while avoiding systemic IL-12 toxicity [79]. A2B530 applies the same Tmod principle to CEA-expressing tumors with HLA-A02 LOH and is being evaluated in colorectal and pancreatic cancers [80,81]. This is particularly important because earlier CEA-directed cellular therapies were limited by colitis, highlighting the need for normal-tissue protection [82]. A2B395 extends the platform into an allogeneic EGFR-directed product with HLA-A02 blocking and beta-2 microglobulin (B2M) knockdown to reduce host-versus-graft rejection [83].

The main limitation of Tmod therapy is patient eligibility. Current HLA-A02-based approaches require HLA-A02 heterozygosity and tumor-specific loss of the HLA-A02 allele. Although HLA-I LOH is relatively common across GI cancers, allele-specific HLA-A02 LOH is substantially less frequent [84]. Prescreening programs such as BASECAMP-1 are therefore essential for identifying eligible patients efficiently [85]. Additional challenges include the need for high-quality tumor sequencing, complex dual-receptor manufacturing, and limited clinical follow-up to date.

### 3.6. Adapter-Mediated and Universal CAR Systems

Adapter-mediated CARs separate antigen recognition from T-cell activation. Instead of recognizing tumor antigens directly, the engineered cell expresses a universal receptor that binds a soluble adapter molecule. The adapter carries both a tag recognized by the CAR and a tumor-binding domain, allowing the same cell product to be redirected toward different antigens by changing the adapter.

The SUPRA CAR system uses a zipCAR expressed on T cells and a zipFv adapter containing a complementary leucine zipper linked to an antigen-binding domain [86]. This design allows retargeting without manufacturing a new CAR-cell product and can implement AND, OR, and NOT logic through combinations of adapters. Activity can also be tuned by adjusting adapter dose or zipper affinity, making the platform highly programmable [49].

The UniCAR platform follows a similar principle, using an anti-tag CAR paired with soluble tagged targeting modules. Because activity depends on the presence of the adapter, cytotoxicity can be pharmacologically controlled and discontinued when the adapter is cleared. UniCAR-FAP targeting modules are especially relevant to GI tumors because they offer a potential way to target stromal components rather than tumor cells alone [58,87]. Related adapter-based platforms, such as RevCAR systems, have also been explored in colorectal cancer using EpCAM-directed adapters [88].

Biotin-binding immune receptor systems illustrate both the promise and the risk of universal CAR designs. In HER2-positive tumor models, biotin-trastuzumab-guided universal CAR-T cells improved spheroid penetration; however, in vivo toxicity occurred due to recognition of endogenous biotin [89]. This finding highlights a central requirement for adapter systems: the tag must be biologically inert, non-cross-reactive with endogenous molecules, and suitable for repeated administration.

For GI cancers, adapter-mediated CARs are attractive because antigen expression may change during treatment. In principle, a universal CAR platform could begin with one target, then switch or add adapters as antigen-negative clones emerge. However, these systems also create new translational challenges, including adapter pharmacokinetics, repeated dosing requirements, immunogenicity of synthetic tags, and the regulatory complexity of evaluating both the cell product and each adapter component.

### 3.7. Summary

Precision antigen targeting has become one of the central engineering priorities for CAR-based therapy in GI oncology. Tandem and bivalent CARs broaden antigen coverage, co-administered and sequential strategies allow modular combination of distinct cell populations, logic-gated circuits improve tumor selectivity, Tmod platforms exploit irreversible tumor-specific genetic loss, and adapter-mediated systems preserve flexibility as the antigen landscape evolves. Each architecture addresses a different aspect of the antigen problem: coverage, specificity, safety, or adaptability.

The most clinically advanced examples, including KD-496, GCC19CART, A2B694, and A2B530, suggest that multi-antigen and logic-gated approaches can improve the therapeutic index of CAR therapy in solid tumors. Nevertheless, the field remains early. Preclinical data across synNotch, LINK, and tandem designs show improved selectivity in xenograft and organoid models, but only KD-496, GCC19CART, and the Tmod platforms have reached patients, all in small, single-arm phase I cohorts still focused on safety rather than efficacy. Where responses occur, most are partial or represent stable disease; none has matched the magnitude of benefit satri-cel achieved in a randomized trial. Sophisticated antigen targeting has not yet outperformed single-target CAR-T in the clinic (Table 1).

## 4. Overcoming Physical and Stromal Barriers

### 4.1. The Desmoplastic Challenge

A dense desmoplastic stroma is a defining feature of gastrointestinal (GI) solid tumors, particularly pancreatic ductal adenocarcinoma (PDAC), where the stromal compartment can exceed the tumor cell mass itself [91]. Activated pancreatic stellate cells generate an extracellular matrix (ECM) rich in collagen, hyaluronan, and other structural proteins that increases tissue stiffness, compresses tumor vasculature, and limits infiltration of cytotoxic lymphocytes, NK cells, and CAR-engineered immune cells [6,92,93,94]. Spatial analyses further show that CD8^+^ T cells are frequently confined to fibroblast-rich stromal regions rather than reaching malignant cells, a pattern associated with poor clinical outcomes [6].

Beyond serving as a physical barrier, cancer-associated fibroblasts (CAFs) actively suppress antitumor immunity by secreting CXCL12, TGF-β, IL-6, and galectin-1, upregulating PD-L1 through STAT3-CCL2 signaling, and promoting hypoxia through hyaluronan-driven vascular compression [52,91,95,96,97]. Together, these mechanisms establish a reinforcing stromal barrier that restricts immune-cell trafficking and function.

Attempts to broadly eliminate the stroma have highlighted its biological complexity. Although stromal depletion can enhance immune infiltration, indiscriminate ablation may also remove tumor-restraining fibroblast populations. Recognition of functionally distinct CAF subsets has therefore shifted the field toward selective stromal remodeling rather than complete stromal depletion [91,98,99].

### 4.2. FAP-Targeted CARs

Fibroblast activation protein (FAP) is the most extensively investigated stromal target for CAR-based therapies because of its high expression on cancer-associated fibroblasts (CAFs) across GI malignancies and limited distribution in normal adult tissues [100,101]. Early preclinical studies demonstrated that FAP-targeted CAR-T cells reduced extracellular matrix deposition, decreased tumor vascular density, and inhibited pancreatic tumor growth [102,103]. However, subsequent work showed that FAP is also expressed on subsets of normal stromal cells. Tran et al. reported recognition of bone marrow stromal cells by FAP-CAR-T cells, resulting in severe cachexia and bone toxicity in mice, highlighting the need for more selective stromal targeting strategies [104].

Recent engineering approaches have therefore focused on improving precision rather than simply increasing cytotoxicity. TALEN-edited hypoimmunogenic universal FAP-CAR-T cells enhanced stromal depletion, CAR-T infiltration, and antitumor activity when administered before mesothelin-directed UCAR-T therapy, with additional benefit observed following PD-1 blockade [54]. Similarly, an IF/THEN-gated dual CAR system linked mesothelin CAR expression to prior FAP recognition, improving antitumor efficacy while reducing on-target, off-tumor toxicity [105]. Sequential stromal remodeling has also proven effective: pretreatment with FAP-CAR-T cells enhanced the efficacy of CLDN18.2-directed CAR-T therapy by depleting CAFs, reducing myeloid-derived suppressor cell recruitment, and improving CD8^+^ T-cell persistence [67]. In parallel, engineering FAP-CAR-T cells to secrete IL-15 promoted a T stem cell memory phenotype associated with greater expansion, persistence, and antitumor activity [106].

Alternative immune-cell platforms are further expanding this strategy. Allogeneic FAP-CAR invariant NKT cells (MiNK-215) efficiently depleted CAFs, increased tumor-specific T-cell infiltration, and generated durable antitumor immunity without detectable off-target toxicity, findings that were validated in organoid models of microsatellite-stable colorectal cancer liver metastases [107]. Likewise, FAP-directed CAR-NK cells effectively targeted both CAFs and tumor cells in preclinical models [108]. Beyond adoptive transfer, targeted lipid nanoparticles delivering FAP-CAR mRNA enabled in vivo CAR generation and achieved antitumor efficacy comparable to retrovirally engineered FAP-CAR-T cells while eliminating the need for ex vivo manufacturing [109].

### 4.3. ECM-Degrading Enzymes

Rather than eliminating stromal cells, an alternative strategy is to engineer CAR-T cells to remodel the extracellular matrix (ECM) directly, thereby improving intratumoral trafficking while preserving stromal components that may retain tumor-restraining functions.

The first proof of concept came from Caruana et al., who demonstrated that ex vivo-expanded T cells lose expression of heparanase (HPSE), the enzyme responsible for degrading heparan sulfate proteoglycans. Restoring HPSE expression in anti-GD2 CAR-T cells enhanced ECM degradation, increased tumor infiltration, and improved antitumor activity in neuroblastoma models [53]. This concept has since been translated to GI malignancies. Zhu S et al. engineered mesothelin-directed CAR-T cells to express HPSE fused to a truncated hepatitis A virus pX domain, enabling pH-dependent extracellular vesicle-mediated enzyme delivery within the acidic tumor microenvironment. Compared with conventional CAR-T cells, this approach increased ECM penetration nearly fourfold, enhanced expression of TRAIL, FasL, and perforin, improved cytotoxicity in two- and three-dimensional colorectal cancer models, and significantly reduced tumor burden while prolonging survival beyond 70 days in HCT116 xenografts without detectable systemic toxicity [110].

To further improve safety, Zheng et al. incorporated synNotch circuits that restricted ECM-degrading enzyme secretion to tumor sites following antigen recognition, thereby enhancing CAR-T infiltration and tumor regression while avoiding detectable systemic toxicity in patient-derived organoid models [111]. In addition to enzyme delivery, CAR architecture itself may influence stromal penetration. Zhang et al. demonstrated that the costimulatory molecule OX40 binds heparan sulfate, increasing CAR-T adhesion, infiltration, persistence, and functional avidity in solid tumor models [112].

Other ECM-remodeling strategies, including hyaluronidase, collagenase, and nattokinase, are also under investigation [28]. Although systemic ECM-targeting agents such as PEGPH20 have shown limited clinical success, localized enzyme delivery and tumor-restricted ECM degradation represent more precise approaches for improving immune-cell infiltration while minimizing systemic toxicity.

### 4.4. Chemokine Receptor Engineering

Crossing the stromal barrier is only the first step; CAR-T cells must also migrate efficiently within the tumor microenvironment. In many solid tumors, trafficking is limited by a mismatch between chemokines produced by the tumor and the chemokine receptors expressed on infused T cells [28,50]. This imbalance is further exacerbated by chemokine pathways such as CCL22/CCR4 and CCL5/CCR5, which preferentially recruit regulatory T cells and myeloid-derived suppressor cells rather than cytotoxic lymphocytes [28].

Chemokine receptor engineering seeks to overcome this limitation by matching CAR-T cells to the chemokine profile of the target tumor. In hepatocellular carcinoma, CXCR2 expression enhanced trafficking and intratumoral accumulation of GPC3-directed CAR-T cells, resulting in improved antitumor activity in CXCR2 ligand-rich tumors [113]. Likewise, CXCR6 conferred superior migration and tumor control compared with CCR2 in mesothelin-targeted CAR-T cells, an effect associated with increased expression of genes involved in immune activation, adhesion, cytoskeletal remodeling, and LFA-1-mediated migration [114]. Combining CCR5 overexpression with IL-12 secretion further enhanced tumor homing while simultaneously promoting macrophage reprogramming in esophageal carcinoma models [115]. Engineering multiple chemokine receptors may further improve trafficking across heterogeneous chemokine gradients; co-expression of CXCR5 and CCR6 increased migration, persistence, and tumor infiltration compared with either receptor alone in HER2-targeted CAR-T cells [116].

An alternative approach is to engineer CAR-T cells to generate their own chemotactic signals. The IL-7/CCL19 (“7 × 19”) platform enhanced immune-cell recruitment, CAR-T infiltration, and antitumor activity in mesothelin- and GPC3-targeted models of hepatocellular and pancreatic cancer, with preliminary clinical findings reporting complete or near-complete tumor regression in patients with advanced HCC and pancreatic cancer [28]. Building on this concept, incorporation of IL-7, IL-15, and CCL19 into B7-H3-directed CAR-T cells further improved immune-cell recruitment and persistence in pancreatic and lung tumor xenografts [117].

Because chemokine expression varies substantially across tumor types and between individual patients, receptor selection should ideally be guided by the chemokine landscape of each tumor. This precision is particularly important because many chemokines are also expressed in normal tissues, raising the possibility of off-target trafficking following forced receptor expression [58].

### 4.5. Regional Delivery

Regional administration offers a practical solution to the trafficking limitations of systemic CAR-T infusion by delivering cells directly to sites of disease. This strategy is particularly relevant in GI malignancies, where peritoneal dissemination is common and remains associated with poor outcomes and limited responses to systemic immunotherapy.

Preclinical studies established the rationale for this approach. Intraperitoneal (I.P.) administration of CEA-targeted CAR-T cells provided superior control of peritoneal tumors compared with intravenous infusion while promoting durable immune protection characterized by increased effector memory T-cell formation, resistance to tumor rechallenge, and systemic antitumor activity against distant lesions accompanied by elevated serum IFNγ levels [118]. Similar findings were reported in gastroesophageal cancer models, where intraperitoneal delivery of mesothelin-targeted M28z1XXPD1DNR CAR-T cells incorporating a PD-1 dominant-negative receptor achieved greater tumor control, prolonged survival, and improved persistence than intravenous administration despite lower cell doses, supporting initiation of an ongoing phase I trial (NCT06623396) [119].

Early clinical studies have supported these observations. In the PC13 trial, hypoxia-responsive CEA-targeted CAR-T cells achieved higher objective response and disease control rates following intraperitoneal than intravenous administration (23.5% vs. 8.0% and 82.4% vs. 68.0%, respectively). Among patients with peritoneal metastases and CEA expression ≥90%, the post hoc objective response rate reached 57.1% (4/7) after regional treatment [120]. Likewise, a phase I study of fast-manufactured Th9/Tc9-polarized CEACAM5-targeted CAR-T cells reported an objective response rate of 57.1%, a disease control rate of 100% in 14 evaluable patients, and a median progression-free survival of 4.7 months despite using approximately one-tenth of the CAR-T cell dose administered in earlier studies [121].

Regional delivery has also shown promise for liver-dominant disease. Hepatic arterial infusion of CEA-targeted CAR-T cells combined with selective internal radiation therapy (HITM-SIR) demonstrated the feasibility of locoregional CAR-T administration and evidence of hepatic responses in colorectal cancer liver metastases [122,123].

Collectively, these studies suggest that regional delivery can increase local CAR-T cell concentrations, reduce the cell dose required for efficacy, and potentially limit systemic toxicity while preserving systemic antitumor activity. Its application, however, remains restricted to anatomically accessible tumors and requires specialized expertise for catheter-based administration.

### 4.6. Hypoxia-Responsive Designs

Hypoxia is a defining feature of desmoplastic GI tumors and can be exploited to improve the specificity of CAR-T cell therapy. Rather than serving solely as a barrier, the hypoxic tumor microenvironment can be harnessed to restrict CAR expression and activation, thereby enhancing tumor selectivity while reducing off-tumor toxicity and tonic signaling.

Two complementary engineering strategies have emerged. The first places CAR expression under the control of hypoxia-response elements (HREs), allowing CAR transcription only in the presence of HIF-1α. Using this approach, Zhu et al. developed the 5H1P-CEA CAR, which maintained minimal CAR expression under normoxic conditions while preserving a less differentiated phenotype, improving oxidative metabolism, reducing exhaustion during ex vivo expansion, and producing more durable antitumor responses than conventional CAR-T cells in patient-derived xenograft models [124]. The second strategy regulates CAR protein stability rather than transcription. He et al. fused the CAR construct to the oxygen-dependent degradation domain (ODD) of HIF-1α to create the HiTA platform, in which CAR proteins undergo rapid degradation under normoxia but are stabilized under hypoxia. In HER2-targeted models, HiTA-CAR-T cells maintained potent antitumor activity while showing negligible CAR expression in normal HER2-positive tissues, including the liver, without detectable systemic toxicity [125].

Clinical translation has begun with the PC13 trial, in which hypoxia-regulated CEA-targeted CAR-T cells demonstrated manageable toxicity in 43 patients with advanced CEA-positive solid tumors treated by either intraperitoneal or intravenous infusion. Grade 1–2 cytokine release syndrome occurred in 76.7% of patients and grade 3 diarrhea in 20.9%, with no severe on-target, off-tumor toxicity despite physiological CEA expression in normal colonic epithelium, providing early clinical support for hypoxia-dependent CAR activation [120].

Recent studies have combined hypoxia sensing with metabolic engineering to further enhance CAR-T function. The Envirotune-CAR-T platform integrated VEGF-derived hypoxia-responsive regulatory elements with overexpression of the glutamine transporter SLC38A2 to improve both hypoxia-induced CAR expression and metabolic fitness under nutrient-deprived conditions [126]. Similarly, increasing endogenous HIF-1α expression using small activating RNA enhanced glycolytic capacity, preserved mitochondrial function, improved infiltration into three-dimensional tumor spheroids, and increased cytotoxicity under hypoxic conditions [127].

Collectively, these studies demonstrate that hypoxia can be exploited as a biological control mechanism rather than simply a feature of the hostile tumor microenvironment. As illustrated by the PC13 trial, combining hypoxia-responsive CARs with complementary strategies such as regional delivery may further improve the safety and efficacy of CAR-T therapy in GI solid tumors.

## 5. Armored Cars and Persistence Engineering

### 5.1. Immunosuppressive Mediators in the GI Tumor Microenvironment

GI tumors suppress CAR-T cell activity through overlapping inhibitory cytokines, immune checkpoints, and metabolic pathways. TGF-β, produced by cancer-associated fibroblasts, regulatory T cells, and tumor cells, inhibits T-cell proliferation, cytokine production, and cytotoxicity while promoting regulatory T-cell differentiation through SMAD2/3 signaling [6,128]. PD-L1 expressed by tumor and myeloid cells further limits CAR-T expansion and effector function through PD-1 engagement [8,129]. Metabolic suppression, including adenosine signaling, lactate accumulation, IDO-mediated tryptophan depletion, arginase activity, and PGE2 production, reinforces these inhibitory pathways [6,8,130]. Together, these mechanisms drive T-cell dysfunction and limited persistence, providing the rationale for armored CAR designs that deliver cytokine support, resist inhibitory signaling, or enhance intrinsic cellular fitness.

### 5.2. Trucks: Cytokine-Armored CAR-T Cells

Fourth-generation CARs (TRUCKs) are engineered to secrete cytokines either constitutively or following antigen recognition, enabling CAR-T cells to improve their own persistence while remodeling the tumor microenvironment [17,131].

IL-15 represents the most clinically advanced cytokine-armoring strategy in GI oncology. In hepatocellular carcinoma, IL-15-armored GPC3 CAR-T cells achieved an objective response rate of 33% and a disease control rate of 66%, whereas conventional GPC3 CAR-T cells produced no objective responses, accompanied by greater in vivo expansion [132]. IL-15 has also enhanced CLDN18.2 CAR-T activity and improved FAP-CAR iNKT-cell function in preclinical GI models [107,133].

Unlike IL-15, IL-12 primarily remodels the tumor microenvironment by repolarizing tumor-associated macrophages and enhancing endogenous immune activation rather than directly supporting CAR-T persistence [131]. To reduce systemic toxicity, newer constructs restrict IL-12 expression to sites of CAR engagement, as illustrated by the NFAT-controlled membrane-tethered IL-12 incorporated into the A2B543 armored Tmod platform [85].

Combinatorial cytokine armoring may further enhance persistence. Co-expression of membrane-tethered IL-15 and IL-21 reduced functional exhaustion and outperformed cytokine alone [134], while similar synergy was reported in GPC3-directed CAR-T cells expressing both cytokines [135]. Likewise, IL-7/CCL19 co-expression combined cytokine support with improved immune-cell recruitment in B7-H3 CAR-T models [117]. Beyond genetic engineering, cytokine-optimized manufacturing using the IL-7/IL-15/IL-21 scaffold HCW9206 enriched T stem cell memory populations, suggesting that some benefits of armoring may also be achieved during ex vivo expansion [136].

The principal limitation of cytokine armoring remains toxicity. IL-15-enhanced GPC3 CAR-T cells increased the incidence of cytokine release syndrome, although events were effectively controlled using IL-1/IL-6 blockade or an inducible caspase-9 safety switch [132].

### 5.3. Dominant-Negative and Switch Receptors

Dominant-negative and switch receptors are designed to neutralize inhibitory signals within the tumor microenvironment by either blocking suppressive pathways or converting them into activating cues.

The dominant-negative TGF-β receptor II (dnTGFβRII) retains the extracellular TGF-β-binding domain but lacks intracellular signaling capacity, thereby preventing SMAD2/3 activation [128]. Clinical proof of concept was established in a phase I trial of PSMA-targeted dnTGFβRII CAR-T cells in metastatic castration-resistant prostate cancer, where one patient achieved a >98% PSA reduction and three others achieved reductions of at least 30%, although one deep responder died following grade 4 cytokine release syndrome complicated by sepsis [137]. In GI-relevant models, incorporation of dnTGFβRII improved the efficacy and persistence of mesothelin-targeted M28z1XXPD1DNR CAR-T cells in gastroesophageal cancer, particularly after intraperitoneal delivery [119], while overcoming CAR-induced TGF-β1-mediated suppression in ROR1-directed pancreatic cancer models [138].

Switch receptors extend this concept by converting inhibitory ligands into stimulatory signals. TB15 links the extracellular domain of TGF-βRII to the IL-15Rα cytoplasmic domain, coupling TGF-β recognition with IL-15-like signaling [139]. Likewise, a TGF-βR/IL-9Rγ domain-swapped receptor triggered STAT5 activation following TGF-β binding and, when combined with membrane-bound IL-2 in CEA-targeted CAR-T cells, enhanced serial killing and antitumor activity across colorectal, gastric, lung, and rectal cancer models [140].

PD-1-based switch receptors similarly redirect inhibitory checkpoint signaling into T-cell activation. The best-characterized design fuses the PD-1 extracellular domain to the CD28 intracellular domain, converting PD-L1 engagement into costimulation [141,142]. Although initially developed in hematologic malignancies, this strategy is increasingly being adapted to GI cancers. A tri-cistronic CLDN18.2 CAR incorporating a PD-1/CD28 switch enhanced antitumor activity in gastric cancer xenografts [143], while similar switch logic has been applied to MUC1-targeted CAR-T cells in cholangiocarcinoma [144]. More recently, PD-1-LAT redirected PD-1 engagement into LAT-dependent proximal signaling and outperformed conventional PD-1/CD28 switches in vivo [145].

### 5.4. Intrinsic Checkpoint Disruption

Gene editing enables inhibitory pathways to be removed directly from CAR-T cells before infusion. PD-1 knockout using CRISPR/Cas9 enhances proliferation, cytokine production, cytotoxicity, and resistance to exhaustion [146]. Clinical feasibility was demonstrated in a phase I trial of mesothelin-targeted CAR-T cells with simultaneous PD-1 and TCR disruption, which reported no dose-limiting toxicities, although stable disease was the best response in only 2 of 15 patients. Preferential persistence of TCR-positive cells suggested that endogenous TCR signaling may still contribute to long-term CAR-T survival in solid tumors [147]. Similarly, PD-1-knockout MUC1 CAR-T cells achieved stable disease in 6 of 9 patients with esophageal cancer, including two patients who survived beyond 24 months [148].

Multiplex genome editing extends this concept by simultaneously addressing several mechanisms of resistance. An allogeneic EGFR-targeted CAR-T product incorporating six gene edits (B2M, CIITA, CD3E, ADORA2A, PDCD1, and TGFBR2) reduced allorejection and graft-versus-host disease while improving resistance to adenosine-, PD-1-, and TGF-β-mediated immunosuppression, resulting in enhanced antitumor activity in EGFR-positive lung tumor xenografts [130].

Checkpoint resistance can also be engineered downstream of receptor signaling. Jin et al. demonstrated that TGF-β impairs CAR-T function by promoting degradation of the transcriptional co-repressor SKI. Expression of SMAD2/3-binding-deficient SKI mutants preserved cytokine production and enhanced tumor cell killing under TGF-β pressure in both liquid and solid tumor models [149].

### 5.5. Metabolic Reprogramming

GI tumors impose profound metabolic stress through hypoxia, nutrient depletion, lactate accumulation, and extracellular acidosis, all of which impair CAR-T-cell proliferation, persistence, and effector function by disrupting glycolytic metabolism and mitochondrial fitness [150].

One strategy is to rewire CAR signaling itself. Sun et al. developed the S71 platform, which incorporates compact IL-2/IL-15 receptor-derived signaling motifs into the CAR intracellular domain, improving mitochondrial function, reshaping tumor-induced metabolic responses, and supporting durable antitumor activity across multiple solid tumor models [151].

Additional approaches target key metabolic pathways. Inhibition of ACAT1 increases free membrane cholesterol, enhances immunological synapse formation, CAR clustering, and cytotoxic granule release. Pharmacologic inhibition with avasimibe improved CAR-T-cell cytotoxicity and IFNγ production, while ACAT1 inhibition restored the function of exhausted HBV- and HCC-specific T cells, suggesting potential applicability to GI-directed CAR-T therapies [52,152]. Fatty acid oxidation (FAO) has likewise emerged as a determinant of long-term persistence. Durable clinical responses have been associated with greater FAO activity and mitochondrial fitness, whereas pharmacologic activation of PPARα/PPARδ and the use of 4-1BB costimulatory domains both promote memory differentiation and sustained antitumor activity [150,153].

Lactate-rich tumor microenvironments represent another therapeutic target. Strategies under investigation include modulation of monocarboxylate transporters (MCT1/MCT4) to improve metabolic resilience and development of the lactic acid-responsive promoter (LARP), which restricts CAR expression to acidic, lactate-rich tumors [28,150,154].

Metabolic regulation is closely linked to epigenetic programming. Maintenance of intracellular acetyl-CoA preserves histone acetylation and cytokine production, whereas manipulation of acetyl-CoA metabolism through ACSS2 overexpression or ACLY inhibition prevented exhaustion and enhanced tumor-specific CD8^+^ T-cell responses [155,156,157,158,159]. Metabolic programming can also be introduced during manufacturing: low-dose decitabine promoted a central memory phenotype and enhanced oxidative phosphorylation, while Envirotune-CAR-T combined hypoxia-responsive regulation with SLC38A2 overexpression to maintain CAR expression and metabolic fitness under nutrient-poor conditions [126,160].

Collectively, these studies suggest that future persistence engineering will extend beyond cytokine armoring and checkpoint resistance to include rational metabolic and epigenetic reprogramming, providing complementary strategies to sustain CAR-T-cell function within the hostile microenvironment of GI solid tumors [150].

### 5.6. Exhaustion-Resistant Phenotypes

CAR-T exhaustion is a stable transcriptional and epigenetic state driven by chronic antigen stimulation, tonic signaling, and the suppressive tumor microenvironment, making resistance to exhaustion a key objective for improving persistence in GI tumors.

One of the best-characterized approaches is c-Jun overexpression. Lynn et al. demonstrated that c-Jun-engineered CAR-T cells exhibited greater expansion, reduced terminal differentiation, and enhanced antitumor activity across multiple tumor models by displacing exhaustion-associated bZIP/IRF transcription factors from AP-1 binding sites [161]. Snyder et al. subsequently showed that PD-1 signaling suppresses c-Jun post-transcriptionally, and that combining c-Jun overexpression with PD-L1 blockade restored c-Jun expression and enabled near-complete tumor clearance [162].

Epigenetic engineering provides an additional strategy to sustain CAR-T function. SUV39H1 inactivation promoted long-term persistence and expanded self-renewing, stem-like populations with reduced dysfunction signatures [163]. Similarly, combined PRDM1 and NR4A3 knockout shifted CAR-T cells toward a TCF1^+^CD8^+^ phenotype and improved antitumor activity, whereas PRDM1 deletion alone induced compensatory NR4A3 upregulation [56]. Triple NR4A knockout likewise enhanced tumor regression in solid tumor models [55].

Disruption of TET2 and DNMT3A represents another promising epigenetic approach. Loss-of-function TET2 mutations were initially associated with remarkable CAR-T expansion and complete remission in chronic lymphocytic leukemia, while experimental disruption of either TET2 or DNMT3A promoted memory-like, less exhausted phenotypes with improved antitumor activity [57,164].

Given the multiple suppressive pressures within GI tumors, durable responses will likely require layered engineering approaches that combine persistence-enhancing strategies with resistance to TGF-β, PD-1 signaling, hypoxia, metabolic stress, and exhaustion (Table 2).

## 6. Allogeneic and Off-the-Shelf Platforms

### 6.1. Manufacturing Bottleneck

The translation of CAR-based therapies in gastrointestinal (GI) solid tumors is limited not only by tumor biology but also by the constraints of autologous manufacturing [10,15]. Autologous CAR-T production requires leukapheresis, ex vivo activation, gene transfer or editing, expansion, quality-control testing, and cryopreservation. This process usually takes two to four weeks and can cost more than $300,000 per patient [25,40]. For patients with rapidly progressive pancreatic, gastric, or metastatic colorectal cancer, this delay can be clinically prohibitive, and progression during vein-to-vein time may prevent infusion altogether [6,126].

Autologous products are also highly variable. Patients with advanced GI cancers are often lymphopenic, exhausted, and heavily pretreated, which compromises starting T-cell fitness and product consistency [6,10]. Manufacturing failure occurs in approximately 10% of hematologic indications and may be higher in heavily pretreated solid tumor populations [40]. Centralized GMP production further limits access, especially in low- and middle-income regions where the GI cancer burden is substantial [2].

Allogeneic and off-the-shelf platforms address these limitations by separating product manufacturing from the individual patient. Healthy donor-derived or stem cell-derived immune cells can be produced in batches, quality controlled, cryopreserved, and administered on demand [165,166]. This model offers shorter treatment timelines, greater product consistency, and improved scalability, making it particularly relevant for GI cancers with aggressive disease kinetics and high global incidence [16,24].

### 6.2. Allogeneic CAR-NK Cells

Natural killer (NK) cells are well suited for allogeneic CAR therapy. Unlike T cells, they do not express a clonotypic T-cell receptor, so allogeneic NK-cell infusion carries minimal risk of graft-versus-host disease (GvHD) [167,168]. NK cells also retain CAR-independent cytotoxicity through NKp30, NKp46, CD16-mediated antibody-dependent cellular cytotoxicity, and NKG2D-mediated stress-ligand recognition (Bauer et al., 1999) [169]. This multimodal killing may help limit antigen escape in heterogeneous GI tumors [168,170].

The clinical feasibility of allogeneic CAR-NK therapy was established by the phase I/II trial of cord blood-derived CD19 CAR-NK cells. Liu et al. reported a 73% response rate in B-cell malignancies, with no cytokine release syndrome (CRS) above grade 1, no neurotoxicity, and no GvHD [171]. Long-term follow-up confirmed durable responses and favorable safety [172]. Lei et al. further supported this platform in refractory large B-cell lymphoma using 4-1BB-costimulated CD19 CAR-NK cells [173].

In GI cancers, CAR-NK platforms are moving into early clinical testing. Wang et al. evaluated membrane-bound IL-15-expressing NKG2D CAR-NK cells in metastatic colorectal cancer (CRC). Treatment was well tolerated, with no treatment-related deaths or serious non-hematologic toxicities. Combination with anti-PD-1 therapy increased peak CAR transgene levels and was associated with overall survival beyond 700 days in two patients [174]. Li et al. also reported intraperitoneal NKG2D CAR-NK infusion in CRC peritoneal metastases, achieving a disease control rate of 33.3% and inducing endogenous CD8^+^ T-cell activation with expansion of cytotoxic CD38^+^HLA-DR^+^ T cells [175].

Preclinical studies have broadened the GI CAR-NK target landscape. PSCA CAR-NK cells showed antitumor activity in orthotopic pancreatic cancer models [176]. Mesothelin CAR-NK cells demonstrated efficacy in CRC patient-derived organoids [177]. GPC3 CAR-NK cells have shown activity in HCC models [178,179], and dual PD-L1/HER2 CAR-NK cells have been designed to counteract antigen heterogeneity through cooperative CAR signaling [180].

The main limitation of CAR-NK therapy remains persistence. NK cells often survive only days to weeks after infusion without cytokine support [167,181]. Current strategies include membrane-bound IL-15, IL-15/IL-15Rα fusion proteins, and immune-evasion engineering [165,174]. Selective HLA knockdown combined with PD-L1 overexpression has also been shown to reduce allogeneic CAR-NK rejection and enhance antitumor activity in xenograft models [182].

### 6.3. iPSC-Derived Platforms

Induced pluripotent stem cells (iPSCs) provide a renewable and clonally defined source for off-the-shelf immune-cell manufacturing. Engineering can be performed at the pluripotent stage, allowing precise transgene insertion, multiplex editing, clonal selection, and release of highly standardized master cell banks [165,183,184]. This approach reduces donor variability and enables production of large numbers of uniform doses from a single engineered clone [165].

Clinical validation of iPSC-derived CAR-NK cells comes from FT596, a CD19 CAR-NK product incorporating a high-affinity non-cleavable CD16 receptor and an IL-15/IL-15Rα fusion protein [165]. In relapsed or refractory B-cell lymphomas, FT596 was well tolerated, with no ICANS, no grade ≥ 3 CRS, and low rates of persistent cytopenias. Responses were seen across lymphoma histologies, including activity after prior autologous CAR-T therapy and an 85% complete response rate in follicular lymphoma [165]. The feasibility of outpatient administration supports the potential scalability of this approach.

For GI solid tumors, iPSC-derived products are being adapted to overcome trafficking, persistence, and immunosuppressive barriers. HER2-targeted iPSC-derived CAR-T cells have been engineered for improved solid tumor activity [185]. Mesothelin-redirected iPSC-derived CAR-NKT cells showed efficacy in orthotopic and metastatic pancreatic cancer models [186]. Mesothelin CAR-IL-15 iPSC-derived NK cells demonstrated enhanced activity against solid tumors, while EpCAM CAR-iPSC-NK cells have also been generated through targeted integration and differentiation [187,188].

The platform also supports complex multi-gene editing. CTX131 incorporates five CRISPR-Cas9 edits: TRAC disruption, B2M knockout, CD70 knockout, TGFBR2 disruption, and Regnase-1 knockout. Together, these edits are designed to prevent GvHD, reduce host rejection, limit fratricide, and resist TGF-β-mediated suppression [189]. This illustrates how iPSC-derived platforms can combine immune evasion, persistence, and tumor microenvironment resistance in a single product.

Key challenges remain. Differentiation protocols are complex and may take four to six weeks. Residual undifferentiated cells must be excluded to prevent teratoma risk, and extensive genomic testing is required after multiplex editing [183,184]. Functional maturation may also differ from primary immune cells, requiring careful optimization before broad clinical translation.

### 6.4. Emerging Effector Cells

Several non-conventional immune lineages are being explored as CAR platforms for GI tumors. These cells may offer advantages over αβ T cells, including reduced alloreactivity, innate tumor recognition, tissue tropism, and microenvironment remodeling.

γδ T cells recognize stress-induced ligands independently of major histocompatibility complex presentation, allowing allogeneic use with low GvHD risk [181]. Vδ1 cells are tissue-resident and relatively exhaustion-resistant, whereas Vγ9Vδ2 cells predominate in peripheral blood and have been widely used for CAR engineering [190]. In the first-in-human phase I study of UTAA06, an allogeneic B7-H3 CAR-Vδ1 T-cell therapy, 10 patients with advanced solid tumors, including gastric, colorectal, and hepatocellular cancers, were treated. No GvHD occurred, CRS was limited to two transient grade 1 events, and no objective responses were observed, likely reflecting limited persistence from subclinical host-versus-graft rejection [191]. PSCA CARs have also been compared across Vδ1, Vγ9Vδ2, and αβ T cells in pancreatic cancer models, showing similar early tumor control but distinct phenotypic and transcriptional programs [192]. ADI-270, a CD70-targeted Vδ1 γδ CAR-T product with dominant-negative TGFβRII, is in phase I/II testing and may inform future GI applications [189].

Invariant natural killer T cells bridge innate and adaptive immunity through a semi-invariant TCR that recognizes lipid antigens presented by CD1d. Their lack of alloreactivity, ability to activate NK cells and cytotoxic T cells, and capacity to reduce suppressive myeloid populations make them attractive off-the-shelf candidates [107,193]. Mesothelin CAR-NKT cells derived from iPSCs targeted orthotopic and metastatic pancreatic cancer in preclinical models [186]. Anti-PSCA CAR-IL-15 iNKT cells promoted pancreatic tumor regression in mice [194]. MiNK-215, a FAP-CAR-IL15 iNKT therapy, remodeled desmoplastic stroma and enhanced antitumor immunity, making it particularly relevant to stromal-rich GI tumors [107].

CAR-macrophages exploit macrophage phagocytosis, antigen presentation, and natural migration into hypoxic and necrotic tumor regions [97,181]. Anti-PSCA CAR macrophages showed potent activity against pancreatic cancer [195]. CAR-M platforms may also remodel extracellular matrix, deplete suppressive tumor-associated macrophages, and cross-prime T-cell responses [196]. However, limited proliferation, possible M2 polarization in the tumor microenvironment, and manufacturing scalability remain major barriers.

### 6.5. Allogeneic CAR-T Cells

Allogeneic CAR-T cells derived from healthy donors offer immediate availability, standardized manufacturing, and potentially superior T-cell fitness compared with autologous products from heavily pretreated patients. Their development is limited by two major barriers: donor TCR-mediated GvHD and host-versus-graft rejection driven by recognition of foreign HLA molecules [197,198].

Gene editing has enabled rational control of these barriers. The standard strategy is TRAC knockout to remove TCR expression and prevent GvHD, combined with B2M knockout to reduce HLA class I expression and limit host T-cell rejection [199,200]. However, HLA class I loss can trigger NK-cell missing-self killing. Additional approaches include HLA-E or single-chain HLA-E insertion to engage NKG2A, or selective HLA-A/HLA-B deletion while retaining matched HLA-C expression [198,200,201].

The most GI-relevant clinical allogeneic CAR-T program is CYAD-101, a non-gene-edited NKG2D-based allogeneic CAR-T product. CYAD-101 co-expresses a truncated CD3ζ peptide with mutated ITAM domains that interferes with endogenous TCR signaling, reducing alloreactivity without genomic editing [202]. In the alloSHRINK phase I trial, CYAD-101 plus FOLFOX in metastatic CRC produced two partial responses and nine cases of stable disease among 15 patients, with median progression-free survival of 3.9 months and no GvHD [6,203]. However, the subsequent KEYNOTE-B79 study combining CYAD-101 with pembrolizumab was placed on FDA clinical hold after two pulmonary toxicity-related deaths, underscoring the need for cautious combination development [6].

ALLO-316, a CRISPR-edited CD70 allogeneic CAR-T product, has shown proof of concept in clear cell renal cell carcinoma. In the TRAVERSE study, disease control reached 81.3% in 16 patients, including one complete response lasting 3 years [189]. Updated data showed an ORR of 20% in CD70-positive tumors and a confirmed ORR of 33% among patients with CD70 expression ≥50% treated at the phase 1b regimen [204]. No GvHD was reported, although three grade 5 treatment-related adverse events occurred [204]. CTX131, a next-generation construct with five gene edits including TGFBR2 and Regnase-1 disruption, is now being evaluated in a phase I/II trial that includes esophageal and pancreatic cancer cohorts [189].

More complex allogeneic designs are emerging. A six-edit EGFR CAR-T product combining B2M, CIITA, CD3E, ADORA2A, PDCD1, and TGFBR2 knockout improved antitumor function in solid tumor xenografts [130]. Universal hypoimmunogenic FAP CAR-T cells depleted tumor stroma and improved infiltration of antigen-targeted CAR-T cells, linking allogeneic engineering with stromal remodeling [54].

Limited persistence remains a central obstacle. Even with gene editing, allogeneic CAR-T cells often decline within two to three weeks after infusion because of residual host rejection [189]. Strategies under study include intensified lymphodepletion, anti-CD52-mediated host T-cell depletion, repeat dosing, and anti-rejection CARs targeting CD70 on activated host alloreactive lymphocytes [205,206].

### 6.6. In Vivo CAR Generation

In vivo CAR generation bypasses ex vivo manufacturing by delivering CAR-encoding nucleic acids directly to immune cells inside the patient. Targeted lipid nanoparticles or viral vectors are used to engineer endogenous T cells, NK cells, or myeloid cells in situ, eliminating leukapheresis, centralized cell culture, and individualized product logistics [166,207].

Lipid nanoparticle platforms are advancing rapidly. FAP-CAR mRNA-loaded targeted LNPs generated CAR T cells in vivo and induced tumor regression in pancreatic cancer models without conventional manufacturing [109]. An immune cell-tropic LNP platform delivering HER2 CAR mRNA generated multilineage “panCAR” immune cells, including T cells, macrophages, dendritic cells, and NK cells. Repeated administration inhibited tumor growth in three syngeneic models and shifted the tumor microenvironment toward immune activation [208]. Beyond individual delivery platforms, a broader conceptual framework has emerged around in vivo T-cell engineering, integrating carrier-level targeting design (ligand architecture, avidity, and endosomal escape), organ-scale exposure (including spleen-tropic delivery as an alternative to conventional liver-directed systems), and expression-level control through lineage-specific promoters and logic-gated payloads to achieve precision without altering biodistribution [209].

Delivery specificity depends strongly on the targeting receptor. Comparative profiling of CD2-, CD4-, CD5-, CD7-, CD8-, and CD4/CD8-targeted LNPs showed that CD7 targeting achieved the most efficient T-cell mRNA delivery, driven more by receptor internalization than receptor abundance [210]. Stable in vivo CAR generation is also being explored using CD7-targeted LNPs carrying minicircle DNA and transposase mRNA, which produced durable CAR-T responses after a single intravenous dose [211].

In vivo CAR-myeloid generation may be particularly relevant for solid tumors. MT-303, an mRNA-LNP therapy encoding a GPC3 CAR, is in first-in-human testing for HCC and is designed to reprogram myeloid cells directly in vivo [212]. An erythrocyte-mediated mRNA delivery platform has also been used to deliver CAR mRNA selectively to CD11b^+^ myeloid cells through splenic homing, producing CAR-myeloid cells that induced complete tumor regression when combined with anti-PD-L1 blockade [213].

Important challenges remain. mRNA-based approaches produce transient CAR expression and may require repeat dosing, although this transient activity may improve safety by functioning as a built-in off switch [207]. Cell-specific delivery remains technically difficult, and repeated LNP administration may be limited by immunogenicity, anti-PEG antibodies, and dose-dependent inflammatory toxicity [166,207]. For GI cancers, in vivo CAR platforms targeting FAP, GPC3, mesothelin, and other GI-relevant antigens could substantially improve scalability and access if delivery specificity and safety are confirmed (Table 3).

## 7. Safety Engineering and Controllability

### 7.1. Safety Imperative

CAR-based therapy in GI solid tumors faces safety challenges that extend beyond the CRS and ICANS profiles seen with hematologic CAR-T products [10,15]. The central concern is on-target, off-tumor toxicity (OTOT), as many GI-relevant targets, including CLDN18.2, mesothelin, CEA, HER2, EpCAM, and GPC3, are also expressed on normal epithelial tissues [6,26]. Fatal cardiopulmonary toxicity after HER2 CAR-T therapy, severe colitis with CEA-directed constructs, and treatment-related deaths in the BPX-601 PSCA GoCAR-T trial illustrate the risks of uncontrolled CAR activity [82,214,215]. CLDN18.2 CAR-T models have also shown gastric mucosal injury at therapeutic doses [38]. These findings have made safety engineering central to GI CAR development, with strategies including affinity tuning, logic gating, suicide switches, pharmacologic control, and transient expression platforms [16,58].

### 7.2. Affinity Tuning

Affinity tuning lowers scFv binding strength so that CAR-T cells preferentially respond to antigen-high tumor cells while sparing antigen-low normal tissues [26]. Reduced-affinity HER2 and EGFR CARs retained activity against antigen-dense tumors while limiting recognition of low-antigen normal cells [216]. Low-affinity HER2 CAR-T cells also achieved systemic efficacy without overt toxicity in solid tumor models [217]. For GI-relevant targets, the optimized hYP218 mesothelin scFv improved tumor infiltration and persistence compared with the original SS1 binder [218].

Logic-gated systems build on the same principle by adding a second layer of specificity. The Tmod platform combines an activating CAR with an inhibitory receptor that recognizes HLA-A02 on normal tissues. Tumor cells with HLA loss of heterozygosity lack the inhibitory ligand and remain susceptible to CAR activation, whereas normal HLA-retaining cells are protected [76,81]. This strategy is now being evaluated in the EVEREST-2 trial for mesothelin-positive pancreatic cancer [219].

### 7.3. Suicide Switches

Suicide switches provide an irreversible emergency brake by eliminating CAR cells after administration of a trigger compound [11,58]. The inducible caspase 9 system uses a modified caspase 9 fused to an FK506-binding protein that dimerizes after rimiducid exposure, rapidly inducing apoptosis and eliminating more than 90% of transduced cells within 30 min [11]. iCasp9 was incorporated into BPX-601, where rimiducid functioned as both a co-stimulatory activator and a safety trigger [215].

Other depletion systems include HSV-TK, which converts ganciclovir into a toxic metabolite and can also serve as a PET reporter, as well as truncated EGFR or CD20 co-expression, which allows antibody-mediated depletion with cetuximab or rituximab [58]. Their shared limitation is that activation permanently destroys the therapeutic product, prompting development of reversible control strategies.

### 7.4. Pharmacologically Controllable Cars

Pharmacologically controllable CARs allow reversible, dose-dependent modulation of activity without eliminating the infused cells. Dasatinib is the simplest example. By inhibiting LCK-mediated CD3ζ/ZAP70 phosphorylation, it produces rapid and reversible CAR silencing while preserving cell viability. In mouse models, short-course dasatinib rescued otherwise fatal CRS [220]. Its limitation is non-selective suppression of all T cells, which restricts prolonged use [58].

Engineered ON-switch CARs require drug exposure to become active. The VIPER platform uses an NS3 protease that continuously cleaves the CAR; NS3 inhibitors such as grazoprevir prevent cleavage and stabilize the intact receptor [221]. OFF-switch CARs use lenalidomide-responsive degron tags to induce proteasomal CAR degradation at subtherapeutic lenalidomide doses [222]. Bidirectional systems combine both concepts. The iONØ-CAR platform is activated by venetoclax and inactivated by lenalidomide, allowing reversible ON/OFF control using two approved drugs [223].

### 7.5. Transient CAR Expression

mRNA-based CAR engineering provides intrinsic safety through non-integrating and self-limited transgene expression [40,207]. Beatty et al. established clinical proof of concept with mesothelin mRNA CAR-T cells in pancreatic cancer, showing antitumor activity and epitope spreading without persistent toxicity (228). NKG2D CAR mRNA-NK cells were also safely tested in metastatic colorectal cancer [224].

This approach is particularly compatible with in vivo CAR generation. Targeted LNP delivery of FAP-CAR mRNA generated CAR T cells in vivo and produced antitumor activity against pancreatic tumors, with transient expression offering a dose-controllable safety margin [109]. Repeated LNP-mRNA delivery has also generated multilineage panCAR immune cells with predictable pharmacokinetics and tumor control [208].

### 7.6. Synthesis

Safety engineering has moved from single safeguards toward layered control systems. Affinity tuning and logic gating improve specificity at the receptor level. Suicide switches provide irreversible emergency elimination. Pharmacologically controllable CARs allow reversible modulation while preserving the therapeutic product. Transient mRNA expression offers built-in self-limitation without permanent genome modification. Increasingly, these mechanisms are being combined within single platforms, as shown by iONØ dual-switch CARs and Tmod logic-gated CARs [58,81]. For GI tumors, where many targets are shared with normal epithelium, such multilayered safety designs are likely to be essential (Table 4).

## 8. Integrative Framework and Future Outlook

### 8.1. Convergence Thesis

Durable clinical responses in GI solid tumors will likely depend on integrating multiple engineering strategies within a single therapeutic platform rather than optimizing any individual component in isolation [6,16,24].

Several observations support this shift. First, although satri-cel represents the most advanced CAR-T product for GI malignancies, the randomized phase II CT041-ST-01 trial reported a median progression-free survival of only 3.25 months, indicating that target selection alone is insufficient for durable disease control [9]. Second, each GI cancer presents distinct biological barriers. PDAC is dominated by stromal exclusion, HCC by a profoundly tolerogenic immune microenvironment, and CRC by marked antigen heterogeneity, making a single engineering solution unlikely to succeed across diseases [6,24,27]. Finally, advances in allogeneic products and in vivo CAR generation now make iterative dosing and modular engineering feasible, allowing armoring strategies, safety switches, and alternative delivery routes to be incorporated into a unified therapeutic design [109,207]. These theoretical advantages come with real engineering and logistical costs. Combining logic gates, cytokine payloads, and safety switches within a single construct increases transgene size beyond the packaging capacity of standard lentiviral vectors in some designs, and single-vector solutions remain an active area of engineering rather than a solved problem [63]. Multiplex-edited and dual-receptor platforms also require more complex manufacturing, which raises production costs and extends vein-to-vein time, a constraint that disproportionately affects patients with rapidly progressive GI malignancies [153]. These trade-offs mean that the most sophisticated preclinical designs are not automatically the most clinically deployable, and platform selection should weigh biological potency against manufacturing feasibility and cost.

The field is therefore moving beyond single-variable optimization toward systems-level engineering, in which antigen selection, CAR architecture, effector cell source, armoring, delivery strategy, combination therapy, and safety mechanisms are designed as complementary components of the same therapeutic platform [29,40].

### 8.2. Disease-Specific Blueprints

Because the biological barriers differ substantially across GI malignancies, engineering strategies should be tailored to each disease rather than applied uniformly.

#### 8.2.1. Pancreatic Ductal Adenocarcinoma (PDAC)

Dense desmoplastic stroma remains the dominant obstacle, restricting immune-cell trafficking while creating a profoundly hypoxic and immunosuppressive niche [6,27]. The most rational approach combines dual-antigen targeting (e.g., mesothelin plus CLDN18.2 or PSCA plus FAP) with stromal remodeling strategies such as FAP depletion or heparanase expression to improve tumor penetration [53,102,226]. Persistence can be supported through IL-15 or IL-21 armoring [132,135], whereas regional intraperitoneal or hepatic arterial delivery may maximize local exposure while limiting systemic toxicity [122]. Additional benefit has been observed when CAR-T cells are combined with IL-12-expressing oncolytic viruses [227]. The BPX-601 experience also highlights the importance of incorporating robust safety controls into PSCA-directed therapies [215].

#### 8.2.2. Hepatocellular Carcinoma (HCC)

GPC3 remains the leading therapeutic target, with early-phase studies demonstrating objective responses and durable disease control [228,229,230]. Future designs are likely to combine GPC3 targeting with persistence-enhancing armoring such as RUNX3 or IL-15/IL-21 [135,229], strategies that overcome shed GPC3-mediated CAR inhibition [43], and additional targets including CD133 or AFP to reduce relapse from stem-like tumor populations [231,232]. Combination with tyrosine kinase inhibitors such as sorafenib or lenvatinib may further improve efficacy through vascular normalization and microenvironment remodeling [233]. Allogeneic GPC3 CAR-NK cells may provide a favorable safety profile because of their lower risk of CRS [178,179].

#### 8.2.3. Gastric and Gastroesophageal Junction (GEJ) Cancer

CLDN18.2 is currently the most clinically validated GI target following the positive phase II satri-cel trial [9]. Future development will likely focus on reducing antigen escape through dual-targeting constructs such as CLDN18.2/NKG2DL [226], integrating CAR-T therapy earlier after first-line chemotherapy [234], incorporating intrinsic checkpoint resistance such as anti-PD-1 scFv secretion [235], and refining companion diagnostic strategies to identify patients with high CLDN18.2 expression [9,37].

#### 8.2.4. Colorectal Cancer (CRC)

CRC remains the most challenging setting because of pronounced antigen heterogeneity [6,24]. Multi-antigen approaches incorporating GCC, CEA, and NKG2D ligands are likely to be more effective than single-target constructs [64,175]. Regional delivery also appears particularly attractive, with hepatic arterial infusion for liver-dominant disease and intraperitoneal administration for peritoneal metastases [118,122]. Early clinical studies suggest that NKG2D CAR-NK cells may additionally stimulate endogenous CD8^+^ T-cell responses [174,175]. The GCC19CART CoupledCAR platform further illustrates how immune amplification strategies may help overcome antigen heterogeneity [64,65].

#### 8.2.5. Biliary Tract Cancer (BTC)

Cholangiocarcinoma and gallbladder cancer remain among the most treatment-resistant GI malignancies, with limited benefit from current systemic therapies and poor long-term survival [6]. Clinical experience with CAR-based therapy is still limited, but several targetable antigens, including MUC1, HER2, mesothelin, EGFR, and GPC3, have been identified across BTC subtypes [24,26]. The strongest preclinical evidence comes from Supimon et al., who showed that MUC1-targeted CAR-T cells incorporating a PD-1/CD28 switch receptor exhibited enhanced cytotoxicity against PD-L1-expressing cholangiocarcinoma cells, highlighting the value of integrating checkpoint resistance into CAR design [144]. Because BTC shares key biological features with PDAC, including extensive desmoplasia, TGF-β-driven immunosuppression, and poor T-cell infiltration [6,8], stromal-targeting and armored CAR strategies developed for pancreatic cancer may also be applicable in this setting. Although clinical evidence remains limited, BTC provides a strong rationale for evaluating next-generation CAR platforms that combine optimized antigen targeting with tumor microenvironment remodeling.

### 8.3. Combination with Existing Therapies

CAR-based therapies are unlikely to replace established treatments but will instead become integrated within multimodal treatment strategies [6,24].

Checkpoint blockade remains one of the most compelling partners because it addresses CAR-T exhaustion within the TME. Both preclinical pancreatic cancer models and early clinical CAR-NK studies support synergy between CAR-based therapies and PD-1 blockade [174,236]. Engineering CARs to secrete anti-PD-1 antibodies or incorporating PD-1 switch receptors may achieve similar benefits while avoiding systemic checkpoint inhibition [144,237].

Radiotherapy represents another promising partner by increasing antigen presentation, promoting immunogenic cell death, and generating tissue-resident memory-like CAR-T phenotypes. Enhanced activity has been demonstrated with proton radiotherapy in pancreatic cancer models [238,239].

Oncolytic viruses provide complementary mechanisms through direct tumor lysis, local immune activation, and improved CAR-T persistence. IL-12-expressing HSV-2 combined with mesothelin CAR-T cells achieved complete tumor elimination in pancreatic cancer xenografts while reducing the required CAR-T dose [227].

Conventional chemotherapy and targeted agents also remain important. Lymphodepletion continues to be essential for CAR engraftment, whereas decitabine priming and sorafenib have shown encouraging synergy through epigenetic reprogramming and TME remodeling, respectively [147,233].

### 8.4. Unresolved Questions

Despite rapid progress, several challenges remain before CAR-based therapies can become routine treatment for GI cancers.

The optimal antigen combinations for each disease remain undefined, and it is unclear whether dual-targeting alone will prevent antigen escape or whether sequential targeting strategies will ultimately be required [41,227]. Direct comparisons between autologous CAR-T, allogeneic CAR-T, CAR-NK, iPSC-derived products, and in vivo CAR generation are also lacking, leaving the optimal effector platform uncertain [167,181].

Additional questions relate to scalability, treatment sequencing, and long-term safety. Whether allogeneic or in vivo platforms can substantially reduce costs while maintaining efficacy remains uncertain [2,24]. Likewise, the optimal integration of CAR therapy with chemotherapy, radiotherapy, checkpoint blockade, and targeted agents has not been defined. Finally, long-term monitoring will be essential to determine the safety of gene-edited products, repeated in vivo mRNA delivery, and chronic antigen engagement in GI tissues [26,38].

Biomarker-guided patient selection requires substantial further refinement, and three distinct challenges must be addressed. First, companion diagnostics for target-antigen expression remain immature. Immunohistochemical thresholds used to define “positive” expression vary across targets and trials, expression is frequently heterogeneous within a single tumor, and single-biopsy sampling may not capture spatial or temporal variation in antigen density. Standardized, target-specific companion diagnostic assays, ideally validated against clinical outcome rather than expression alone, will be needed before biomarker-guided enrollment can be applied consistently across GI CAR platforms.

Second, HLA-LOH screening, essential for Tmod eligibility, adds a further layer of complexity. Allele-specific HLA-A02 loss follows distinct patterns by tumor type and is substantially less common than HLA class I LOH overall [84], so prescreening programs such as BASECAMP-1 require high-quality tumor sequencing and dedicated infrastructure that may not be broadly available outside specialized centers.

Third, tumor-intrinsic features should guide platform selection itself, not only patient eligibility. Antigen density and expression homogeneity may favor single-target or affinity-tuned CARs, whereas heterogeneous or low-density expression may better support multi-antigen or logic-gated architectures. Tumors with dense desmoplastic stroma, such as PDAC, may benefit preferentially from stromal-remodeling or regional-delivery strategies, while a profoundly immunosuppressive microenvironment may require armored or persistence-enhanced constructs regardless of antigen selection. No prospective framework currently integrates these features into a unified selection algorithm; developing one is a necessary next step toward personalized CAR-based therapy in GI oncology (Table 5).

## 9. Conclusions

CAR-based therapy has transformed the management of hematologic malignancies, but translating this success to gastrointestinal solid tumors requires overcoming a far more complex biological landscape. The core synthesis of this review is that antigen heterogeneity, stromal exclusion, immunosuppressive signaling, and T-cell exhaustion do not operate in isolation, and therefore, cannot be solved by single-variable optimizations. This work’s integrated barrier-to-engineering framework demonstrates that clinical efficacy relies on the synchronized pairing of complementary synthetic biology tools [6,10,26].

Multi-antigen and logic-gated CARs aim to improve tumor specificity, stromal remodeling and cytokine armoring enhance persistence within hostile microenvironments, while allogeneic platforms, iPSC-derived products, and in vivo CAR generation promise to expand access through scalable manufacturing [54,109,165,172,208]. At the same time, advances in safety engineering including pharmacologically controlled CARs, suicide switches, and transient mRNA expression are improving the feasibility of targeting epithelial malignancies where on-target, off-tumor toxicity remains a major concern [58,225].

The randomized satri-cel trial provides the first convincing clinical evidence that CAR-T therapy can improve outcomes in a GI solid tumor [9]. Equally important, however, it demonstrates that targeting a single antigen alone is unlikely to produce durable disease control. Future progress will depend on integrating complementary engineering strategies into disease-specific therapeutic platforms that simultaneously address antigen escape, stromal barriers, immune suppression, trafficking, persistence, manufacturing, and safety.

The next generation of CAR therapies will likely be defined not by a single technological advance, but by the rational combination of multiple engineering innovations within programmable, biomarker-guided cellular therapies. As synthetic biology, genome editing, and scalable manufacturing continue to mature, this integrated approach has the potential to establish CAR-based cell therapy as a clinically meaningful treatment modality across GI solid tumors [2].

## Figures and Tables

**Table 1:** Multi-antigen and logic-gated CAR architectures for GI solid tumors. Abbreviations: CAR, chimeric antigen receptor; CLDN18.2, claudin 18.2; CRC, colorectal cancer; DLT, dose-limiting toxicity; FAP, fibroblast activation protein; GI, gastrointestinal; GPC3, glypican-3; HCC, hepatocellular carcinoma; HLA, human leukocyte antigen; LOH, loss of heterozygosity; mCRC, metastatic colorectal cancer; memIL-12, membrane-tethered interleukin-12; MSLN, mesothelin; NK, natural killer; OTOT, on-target off-tumor toxicity; PDAC, pancreatic ductal adenocarcinoma; PFS, progression-free survival; scFv, single-chain variable fragment; TME, tumor microenvironment.

Architecture	Mechanism	Logic	GI-Relevant Example(s)	Key Finding	Advantages	Limitations	Clinical Stage	References
**Tandem (bivalent) CAR**	Two scFvs; either antigen activates signaling	OR	KD-496 (CLDN18.2 + NKG2DL); GPC3/CD133 (HCC); PD-L1/CLDN18.2 (gastric)	KD-496: responses in refractory GI cancers, minimal toxicity; GPC3/CD133: improved control vs. monospecific CARs	Single construct; broader antigen coverage	Large transgene; tonic signaling risk	Phase I (KD-496); preclinical (others)	[28,59,61]
**Co-administered CARs**	Separate CAR-cell products, co-infused	OR (population level)	GCC19CART (GCC + CD19) in mCRC	Dose-dependent responses; encouraging PFS	Modular; independent dose optimization	Dual manufacturing; regulatory complexity	Phase I/II	[64,65,66]
**Sequential CAR therapy**	Stromal-targeting CAR before tumor-targeting CAR	Sequential	FAP CAR-T → CLDN18.2 CAR-T (PDAC)	Stromal depletion enhanced CLDN18.2 efficacy and CD8^+^ persistence	Addresses stromal barrier before tumor targeting	Two manufacturing cycles; higher cost	Preclinical	[67]
**SynNotch IF–THEN**	Antigen A induces expression of CAR against antigen B	IF–THEN	GPC3-synNotch → CD147 CAR-NK (HCC); HER2-synNotch → MSLN CAR-T	Improved specificity while reducing tonic signaling	Spatially restricted; preserves stem-like phenotype	Delayed, transcription-dependent activation	Preclinical	[11,70,89]
**AND-gate (LINK)**	Split signaling via LAT/SLP-76; both antigens required	AND	Proposed for CEA + CLDN18.2 or EpCAM + GPC3	High antigen discrimination (preclinical)	Rapid; reversible; highly specific	No GI validation; requires dual-antigen expression	Preclinical	[72]
**AND-gate (OriC613)**	Affinity-tuned CAR requiring MSLN + CLDN18.2	AND	OriC613 (gastric cancer)	Selective killing of double-positive cells; spares CLDN18.2-only	Improved safety for CLDN18.2 targeting	Misses single-antigen-positive tumors	Preclinical	[73]
**Inhibitory (NOT-gate) CAR**	Activating CAR + inhibitory receptor for normal-tissue antigen	NOT	HER2 CAR + HLA-A02/LIR-1 inhibitory CAR	Protected HLA-A02^+^ cells (3D models)	Reduces on-target, off-tumor toxicity	Incomplete inhibition; signaling kinetics; cis-binding	Preclinical	[74,75]
**Tmod platform**	Activating CAR + HLA-A02 blocker; restricted to LOH^+^ tumors	NOT (LOH-based)	A2B694 (MSLN); A2B530 (CEA); A2B543 (MSLN + memIL-12)	Early evidence of infiltration; no DLTs	Exploits stable genomic LOH; resistant to antigen downregulation	Requires HLA-A02 heterozygosity + tumor LOH; extensive prescreening	Phase I	[79,81,90]
**Adapter-mediated (SUPRA, UniCAR, RevCAR)**	Universal CAR binds adapter carrying tumor ligand	Programmable (OR/AND/NOT)	UniCAR-FAP; RevCAR-EpCAM; biotin-trastuzumab	Retargetable without a new CAR product	Flexible targeting; titratable; sequential antigen switching	Repeated dosing; PK variability; regulatory complexity	Preclinical	[86,87,88,89]

**Table 2:** Armored CAR and persistence engineering strategies with GI cancer relevance. Abbreviations: CAR, chimeric antigen receptor; CRC, colorectal cancer; DCR, disease control rate; dnTGFβRII, dominant-negative transforming growth factor-β receptor II; EGFR, epidermal growth factor receptor; GEJ, gastroesophageal junction; GPC3, glypican-3; HCC, hepatocellular carcinoma; I.P., intraperitoneal; MSLN, mesothelin; mbIL-2, membrane-bound interleukin-2; memIL-12, membrane-tethered interleukin-12; NFAT, nuclear factor of activated T cells; ORR, objective response rate; OS, overall survival; PDAC, pancreatic ductal adenocarcinoma; TCF1, T-cell factor 1; TGF-β, transforming growth factor-beta.

Strategy	Mechanism	GI-Relevant Example	Key Finding	Reference
IL-15 armoring	Autocrine survival signal	GPC3 CAR-T in HCC	DCR 66%, ORR 33% versus 0% without IL-15	[132]
IL-15/IL-21 co-expression	Synergistic anti-exhaustion signaling	GPC3 CAR-T in HCC	Superior expansion versus single cytokine	[134,135]
IL-7/CCL19 co-expression	Survival support and chemokine recruitment	B7-H3 CAR-T in PDAC	Enhanced infiltration and efficacy	[121]
dnTGFβRII	Competitive TGF-β blockade	MSLN CAR-T in GEJ cancer	Superior efficacy with I.P. delivery	[119,137]
TGF-βR/IL-15Rα ICR	Converts TGF-β into IL-15-like signaling	EGFR CAR-T in solid tumors	Enhanced persistence in high-TGF-β tumors	[139]
TGF-βR/IL-9Rγ DSCR plus mbIL-2	Converts TGF-β into STAT5 activation	CEA CAR-T in CRC and gastric cancer	8 versus 5 rounds of serial killing	[140]
PD-1/CD28 switch	Converts PD-L1 into co-stimulation	CLDN18.2 CAR-T in gastric cancer	Sustained tumor regression	[143]
PD-1-LAT switch	Redirects PD-1 to LAT proximal signaling	HER2 CAR-T in solid tumors	Outperformed PD-1/CD28 in vivo	[145]
PD-1 knockout	Permanent checkpoint removal	MUC1 CAR-T in esophageal cancer	OS > 24 months in 2/9 patients	[148]
Multiplex editing	Simultaneous multi-barrier resistance	EGFR allogeneic CAR-T	Enhanced effector function	[130]
SKImut2/3 expression	Prevents TGF-β-induced SKI degradation	CAR-T cells in liquid and solid tumors	Superior killing under TGF-β pressure	[149]
c-Jun overexpression	Displaces exhaustion AP-1 factors	Multiple solid tumors	Exhaustion resistance across five models	[161]
c-Jun plus anti-PD-L1	Synergistic exhaustion reversal	Lung cancer models	Near-complete tumor clearance	[162]
PRDM1/NR4A3 dual knockout	Disrupts exhaustion feedback	Prostate cancer CAR-T	Shift toward TCF1^+^CD8^+^ phenotype	[56]
SUV39H1 inactivation	H3K9me3 epigenetic reprogramming	Lung and disseminated solid tumors	Long-term stem-like persistence	[163]
Decitabine priming	DNA methylation reprogramming	CAR-T cells	Enhanced central memory phenotype	[160]
S71 modular CAR	IL-2/IL-15R motifs in CAR intracellular domain	Multiple solid tumors	Improved mitochondrial function	[151]

**Table 3:** Allogeneic and off-the-shelf CAR-based platforms for GI solid tumors. Abbreviations: ADCC, antibody-dependent cellular cytotoxicity; CAR, chimeric antigen receptor; ccRCC, clear cell renal cell carcinoma; CRC, colorectal cancer; CRS, cytokine release syndrome; CTL, cytotoxic T lymphocyte; DC, dendritic cell; FAP, fibroblast activation protein; GI, gastrointestinal; GPC3, glypican-3; GvHD, graft-versus-host disease; HCC, hepatocellular carcinoma; HLA, human leukocyte antigen; HvG, host-versus-graft; iNKT, invariant natural killer T; iPSC, induced pluripotent stem cell; KO, knockout; LNP, lipid nanoparticle; mCRC, metastatic colorectal cancer; MHC, major histocompatibility complex; MSLN, mesothelin; NK, natural killer; PB, peripheral blood; PSCA, prostate stem cell antigen; TCR, T-cell receptor; tLNP, targeted lipid nanoparticle; TME, tumor microenvironment.

Platform	Cell Source	Key Advantages	Key Limitations	GI-Relevant Examples	Clinical Stage	References
Allogeneic CAR-NK	Cord/peripheral blood NK cells; NK-92 line	Low GvHD risk; CAR-dependent and innate killing; low CRS	Limited persistence; TME suppression; variable expansion	NKG2D CAR-NK ± anti-PD-1 in mCRC; PSCA CAR-NK in pancreatic cancer; GPC3 CAR-NK in HCC	Phase I in mCRC	[174,175,176,192]
iPSC-derived CAR-NK	Clonal iPSC master cell bank	Unlimited supply; batch uniformity; multiplex engineering	Complex differentiation; teratoma risk; maturation variability	MSLN CAR-IL15 iPSC-NK; EpCAM CAR-iPSC-NK	Phase I in hematologic cancer; GI preclinical	[165,187,188]
CAR-γδ T cells	Expanded Vδ1 or Vγ9Vδ2 donor cells	MHC-independent recognition; low GvHD risk; tissue residency; innate and adaptive killing	Limited clinical data; host rejection; subset-specific optimization	B7-H3 CAR-Vδ1T in GI tumors; PSCA CAR-Vδ1 in pancreatic cancer	Phase I in solid tumors including GI cancers	[191]
CAR-iNKT cells	Peripheral blood-expanded or iPSC-derived iNKT cells	No alloreactivity; CD1d-mediated immunomodulation; activation of NK, CTL, and DC compartments	Rare starting population; expansion challenges; limited GI clinical data	MSLN CAR-NKT in pancreatic cancer; FAP-CAR-IL15 iNKT; PSCA CAR-iNKT	Preclinical/Phase I	[107,192,194]
CAR-macrophages	Peripheral blood monocytes or iPSC-derived macrophages	Tumor infiltration; phagocytosis; antigen presentation; TME remodeling	Limited proliferation; M2 polarization risk; manufacturing challenges	PSCA CAR-M in pancreatic cancer; NKG2D CAR-M in HCC	Preclinical	[195,196]
Gene-edited allogeneic CAR-T	Healthy donor peripheral blood T cells	Standardized product; immediate availability; improved donor T-cell fitness; multiplex editing	GvHD risk; host rejection; limited persistence; safety signals	CYAD-101 in mCRC; CTX131 in esophageal/pancreatic cancer cohorts; FAP CAR-T	Phase I in mCRC and ccRCC; GI cohorts enrolling	[202]
In vivo CAR generation	Endogenous T, NK, and myeloid cells	No ex vivo manufacturing; scalable; multilineage engineering; transient expression may improve safety	Transient CAR expression; repeat dosing; off-target transfection; LNP immunogenicity	FAP-CAR tLNP in pancreatic cancer; GPC3 in vivo CAR for HCC; HER2 panCAR	Phase I in HCC; pancreatic preclinical	[109,208,212]

**Table 4:** Safety engineering strategies for CAR-based cell therapy in GI solid tumors. Abbreviations: ADCC, antibody-dependent cellular cytotoxicity; CAR, chimeric antigen receptor; CRC, colorectal cancer; CRS, cytokine release syndrome; GI, gastrointestinal; HLA, human leukocyte antigen; iCasp9, inducible caspase 9; ICANS, immune effector cell-associated neurotoxicity syndrome; LNP, lipid nanoparticle; LOH, loss of heterozygosity; MSLN, mesothelin; mRNA, messenger RNA; OTOT, on-target off-tumor toxicity; PK, pharmacokinetics; PSCA, prostate stem cell antigen; scFv, single-chain variable fragment; tEGFR, truncated epidermal growth factor receptor.

Strategy	Mechanism	Reversibility	Key Advantages	Key Limitations	GI-Relevant Examples	References
Affinity tuning	Reduced scFv affinity favors antigen-high over antigen-low tissues	Intrinsic	No added module; preserves high-density activity	May reduce activity against antigen-low subclones	Low-affinity HER2 CAR-T; hYP218 MSLN CAR-T	[216,217,218]
Logic-gated CARs/Tmod	Activating CAR + inhibitory receptor; blocked when HLA ligand present	Intrinsic	Tumor-selective; protects HLA-retaining normal tissues	Requires HLA typing; limited to LOH tumors	MSLN Tmod CAR-T in pancreatic cancer	[76,81,219]
tEGFR/CD20 depletion	Surface markers enable antibody-mediated depletion using cetuximab or rituximab	Irreversible	Use approved antibodies; enables cell tracking	Depending on host effector function	Multiple CAR constructs	[58]
Dasatinib OFF-switch	LCK inhibition blocks CD3ζ/ZAP70 signaling	Reversible	Immediate; titratable; uses approved drug	Non-selective T-cell suppression	CRS/ICANS rescue strategy	[220]
ON-switch CAR/VIPER	NS3 protease cleaves CAR; NS3 inhibitors stabilize the receptor	Reversible	Drug-dependent activity; compatible with OFF-switch logic	Requires continued drug exposure; added construct complexity	Preclinical solid tumor models	[221]
Degron OFF-switch	Lenalidomide induces proteasomal CAR degradation	Reversible	Rapid; active at subtherapeutic doses	Potential lenalidomide off-target effects	Preclinical models	[222]
Dual ON/OFF iONØ-CAR	Venetoclax activates CAR; lenalidomide induces degradation	Reversible, bidirectional	Full bidirectional control with approved drugs	Complex construct; early-stage evidence	Preclinical models	[223]
mRNA transient expression	Non-integrating mRNA decays over time	Self-limiting	No genomic integration; predictable pharmacokinetics; inherent safety	Limited persistence; repeated dosing often required	MSLN mRNA CAR-T in pancreatic cancer; NKG2D mRNA CAR-NK in CRC; FAP-CAR LNP in vivo	[109,224,225]

**Table 5:** Disease-specific engineering blueprints for CAR-based cell therapy in GI solid tumors. Abbreviations: AFP, alpha-fetoprotein; BTC, biliary tract cancer; CAR, chimeric antigen receptor; CEA, carcinoembryonic antigen; CLDN18.2, claudin 18.2; CRC, colorectal cancer; EGFR, epidermal growth factor receptor; EpCAM, epithelial cell adhesion molecule; FAP, fibroblast activation protein; GCC, guanylyl cyclase C; GEJ, gastroesophageal junction; GI, gastrointestinal; GPC3, glypican-3; HCC, hepatocellular carcinoma; MSLN, mesothelin; MUC1, mucin 1; NK, natural killer; NKG2DL, NKG2D ligand; NKT, natural killer T; OTOT, on-target off-tumor toxicity; PD-1, programmed cell death protein 1; PDAC, pancreatic ductal adenocarcinoma; PSCA, prostate stem cell antigen; RUNX3, RUNX family transcription factor 3; TGF-β, transforming growth factor-beta; VEGF, vascular endothelial growth factor.

Feature	PDAC	HCC	Gastric/GEJ	CRC	BTC	References
Primary targets	Mesothelin, CLDN18.2, PSCA	GPC3, CD133, AFP	CLDN18.2, HER2, MSLN	GCC, CEA, NKG2DL, EpCAM	MUC1, HER2, MSLN, EGFR, GPC3	[6,9,144]
Dominant barrier	Dense desmoplastic stroma	Immunosuppressive hepatic microenvironment	Peritoneal spread; checkpoint signaling	Antigen heterogeneity; liver metastatic niche	Desmoplastic stroma; TGF-β-driven immunosuppression	[6,27]
Preferred targeting strategy	MSLN + FAP; CLDN18.2 + NKG2DL	GPC3 + CD133; GPC3 + AFP	CLDN18.2 + NKG2DL; CLDN18.2 + MSLN	GCC + CD19; CEA + NKG2DL	MUC1 + PD-1 switch; MSLN + FAP	[54,64,226]
Priority engineering	IL-15/IL-21; heparanase	RUNX3; IL-15/IL-21	Anti-PD-1 scFv; IL-15	Chemokine receptors; IL-12	dnTGFβRII; PD-1/CD28 switch; IL-15 armoring	[132,133,229]
Preferred delivery	Intraperitoneal; hepatic arterial	Hepatic arterial; intratumoral	Intravenous; intraperitoneal	Hepatic arterial; intraperitoneal	Hepatic arterial; intravenous	[119,122]
Promising effector platform	Armored autologous CAR-T; allogeneic CAR-NKT	Autologous CAR-T; allogeneic CAR-NK	Autologous CAR-T	Allogeneic CAR-NK; autologous CAR-T	Armored autologous CAR-T	[126,179,226]
Combination partner	IL-12 oncolytic viruses; anti-PD-1	Sorafenib/lenvatinib; anti-PD-1	Sequential post-first-line therapy; anti-PD-1	Anti-VEGF; radiotherapy	Anti-PD-1; gemcitabine/cisplatin backbone	[227,233,237]
Safety priority	Pharmacologic switches; affinity tuning	Affinity tuning; hepatic monitoring	OTOT mitigation; companion diagnostics	Logic-gated CARs; regional delivery	Affinity tuning; hepatic monitoring	[43,81,215]
Most advanced clinical evidence	Phase I	Phase I/Ib	Randomized phase II	Phase I	Preclinical	[65,226,240]
Key unresolved question	Durable stromal remodeling	Balancing efficacy with hepatic safety	Long-term durability	Optimal antigen combinations	Target validation in clinical cohorts	[6,26,27]

## Data Availability

No new data were created or analyzed in this study. Data sharing is not applicable to this article.
